# Optimizing Traditional Cropping Systems Under Climate Change: A Case of Maize Landraces and Bambara Groundnut

**DOI:** 10.3389/fsufs.2020.562568

**Published:** 2020-10-22

**Authors:** Vimbayi G. P. Chimonyo, Eranga M. Wimalasiri, Richard Kunz, Albert T. Modi, Tafadzwanashe Mabhaudhi

**Affiliations:** 1 Centre for Transformative Agricultural and Food Systems, School of Agricultural, Earth & Environmental Sciences, University of KwaZulu-Natal, Pietermaritzburg, South Africa; 2 Crops for the Future Research Centre, Semenyih, Malaysia; 3 Centre for Water Resources Research, School of Agricultural, Earth & Environmental Sciences, University of KwaZulu-Natal, Pietermaritzburg, South Africa

**Keywords:** climate change adaptation, climate change impacts, food and nutrition security, multicropping, neglected and underutilized crops, resilience, water use

## Abstract

Traditional crop species are reported to be drought-tolerant and nutrient-dense with potential to contribute to sustainable food and nutrition security within marginal production systems under climate change. We hypothesized that intercropping maize landraces (*Zea mays* L.) with bambara groundnut (*Vigna subterranea* (L.) Verdc.), together with optimum management strategies, can improve productivity and water use efficiency (WUE) under climate change. Using an ex-ante approach, we assessed climate change impacts and agronomic management options, such as plant ratios, and plant sequences, on yield and WUE of intercropped maize landrace and bambara groundnut. The Agricultural Production Systems sIMulator (APSIM) model was applied over four time periods; namely past (1961–1991), present (1995–2025), mid-century (2030–2060) and late-century (2065–2095), obtained from six GCMs. Across timescales, there were no significant differences with mean annual rainfall, but late century projections of mean annual temperature and reference crop evaporation (ET_0_) showed average increases of 3.5°C and 155mm, respectively. By late century and relative to the present, the projected changes in yield and WUE were −10 and −15% and 5 and 7% for intercropped bambara groundnut and maize landrace, respectively. Regardless of timescale, increasing plant population improved yield and WUE of intercropped bambara groundnut. Asynchronous planting increased yield and WUE for both maize landrace (5 and 14%) and bambara groundnut (35 and 47%, respectively). Most significant improvements were observed when either crop was planted 2–3 months apart. To reduce yield gaps in intercrop systems, low-cost management options like changing plant populations and sequential cropping can increase yield and WUE under projected climate change. To further increase sustainability, there is a need to expand the research to consider other management strategies such as use of other traditional crop species, fertilization, rainwater harvesting and soil conservation techniques.

## Introduction

Sub-Saharan Africa has a dualistic food system with the formal system taking a more national focus, and also focused on a few strategic crops while the informal system supports local food systems, which support household food and nutrition security (Tcoli, 2016; Mabhaudhi et al., 2019a). While several nations are food secure at a national level, household food insecurity remains problematic with an estimated 821 million people currently food insecure and malnourished (Abegaz, 2018; Gashu et al., 2019; Xie et al., 2019). Most of these people rely on agriculture as their mainstay; thus, the importance of agriculture within these communities provides an opportunity to improve food and nutrition security, reduce poverty, and enhance rural economic development [New Partnership for Africa’s Development (NEPAD), 2014]. However, current crop yields are low and challenged by worsening land degradation, especially declining soil fertility (Ukeje, 2010; Rippke et al., 2016; Badu-Apraku and Fakorede, 2017), and low water use efficiency (WUE) (Mabhaudhi et al., 2018b; O’Leary et al., 2018; Nouri et al., 2019). Furthermore, climate variability and change are adversely affecting productivity through increased incidences and intensity of droughts (Mpandeli et al., 2018; O’Leary et al., 2018; Nhamo et al., 2019). There is consensus that rural agricultural systems must increase resource use efficiencies and adopt strategies to adapt to climate risk (Isaacs et al., 2016; Matthews and McCartney, 2018).

A considerable amount of literature depicts the adoption of improved technologies such as the use of high yielding, improved crop varieties (Hammer et al., 2014; Ran et al., 2017; Mabhaudhi et al., 2019a). However, marginalized farmers have experienced several challenges when trying to adopt conventional farming practices. Chief among these include inadequate access to agrochemicals, loss in agro-biodiversity and an increase in the vulnerability of the system to climate risk (Mabhaudhi et al., 2019b; Malik and Chaudhary, 2019). The low adoption and consequent challenges have partly contributed to the widening gaps in food and nutrition security (Midega et al., 2015; Mrema et al., 2018). Within the context of marginal systems, agriculture needs to sustainably contribute to food and nutrition security and rural economic development, while reducing negative impacts on the environment or improving the environment (van Ittersum et al., 2016). Demand for more sustainable agriculture, which is less dependent on external inputs and better suited to marginal environments, has revived interest in traditional systems (Keatinge et al., 2015; Govender et al., 2016; Saharan et al., 2018). In line with this, there is a renewed focus on the inclusion of neglected and underutilized crops (NUS) as alternative crop choices in marginal cropping systems (Mabhaudhi et al., 2019a).

Neglected and underutilized crops, also referred to as underutilized indigenous and traditional crops, are defined as “plant species that are part of more substantial biodiversity, were once popular (in and out of their centers of diversity), and are neglected by users and research but remain relevant in the regions of their diversity” (Dansi et al., 2012). They are associated with high nutritional value, adaptation to marginal soils, and tolerance to drought and heat stresses (Slabbert et al., 2004; Chibarabada et al., 2015; Chimonyo et al., 2016a; Hadebe et al., 2017; Mabhaudhi et al., 2017). They often require fewer inputs such as fertilizer and agrochemicals, as they are also tolerant of several pests and diseases (Mabhaudhi et al., 2019a). Their nutritional attributes and adaptability make them suitable crops for promotion in marginal areas where poverty and food and nutrition insecurity remain high; however, their contribution to mainstay agriculture remains low (Massawe et al., 2016). As is reflected by their name, the potential of underutilized crops has not yet been fully harnessed, but most of them contribute to diversification and resilience of agroecosystems. Therefore, they have the potential for future agriculture under adverse agro-climatic conditions (Padulosi et al., 2011). Many proponents of modern agriculture and the Green Revolution have discouraged their continued production, highlighting low productivity and resource use efficiencies (Tokatlidis and Vlachostergios, 2016; Missio et al., 2018). For example, water use efficiency of bambara groundnut was reported to be 0.45 kg ha^−1^ mm^−1^ compared to 0.89 kg ha^−1^ mm^−1^ for groundnut (Chibarabada et al., 2017), while landrace sorghum varieties had 20% less WUE relative to hybrid varieties (Hadebe et al., 2019). However, the argument is to not promote them as replacement crops for high yielding major crops, but as complementary crops (Mabhaudhi et al., 2019a), especially in marginal areas where the major crops may not perform well (Massawe et al., 2016). Within these areas, NUS have potential to contribute to improving rural livelihoods and may be “better bet” technologies; however, this potential remains largely untapped due to limited information detailing their genetic, eco-physiological and agronomic performance (Chivenge et al., 2015). It is against this backdrop we hypothesize that, by optimizing resource use, yields of NUS can be sustainably increased. Intercropping involves growing of two or more crops simultaneously or overlapped on the same piece of land, which can sustainably increase WUE (Martin-Guay et al., 2018).

In this study, we hypothesize that intercropping a maize landrace (*Zea mays* L.) with bambara groundnut (*Vigna subterranea* (L.) Verdc.) is beneficial because the latter’s smaller canopy offers little competition to the cereal crop (Saxena et al., 2018). As a legume, bambara groundnut also fixes atmospheric nitrogen. It contributes to soil fertility (Sprent et al., 2010), and the low cost of bambara groundnut seed makes it an exemplar crop for enhancing food and nutrition security within cereal producing households (Muhammad et al., 2016; Mayes et al., 2019). While traditional cropping systems featured multicrops (Muzari et al., 2012), intercropping maize with bambara groundnut is no longer a common practice. Little information is known about crop interaction and the impacts of climate variability and change on productivity and water productivity. While intercropping, in general, could be considered positive in terms of yield (Martin-Guay et al., 2018), the performance of each crop in an intercrop system is determined by the interaction between different crops and the availability of resources. With the impacts of climate variability and change, adapting agronomic management in response to changing resources can allow for sustainable intensification of the traditional cropping systems through improved resource use efficiency. Using an ex-ante approach in APSIM, the current study assessed the productivity and water use of a maize landrace - bambara groundnut intercrop under changing climate and in response to different management options. APSIM has been used widely to study impacts of climate change on crop growth and productivity across Africa (Beveridge et al., 2018; Duku et al., 2018; Xiao et al., 2020). However, its application for studying intercrop systems remains scanty, with no known research on its application for climate change studies.

## Materials and Methods

### Study Area

The study area was the University of KwaZulu–Natal’s Ukulinga Research Farm (29° 40′S; 30° 24′E; 809 m a.s.l.). Ukulinga Research Farm is classified as semi-arid with 77% of the mean annual rainfall of 750 mm received mostly between October and April. The summer months are warm to hot, with an average temperature of 26.5°C (Kunz et al., 2015). Soil textures are characterized as predominantly clay to clay loam and are moderately shallow, ranging from 0.6 to 0.8 m (Chimonyo et al., 2016a).

### APSIM Maize – Bambara Groundnut Intercrop Model

#### Brief Description of the APSIM Model

The APSIM version 7.10 is a daily time step, field-scale multi-year, a multi-crop model that provides an analytical tool for assessing the impacts of climate, soil factors and farming management on cropping system production (Holzworth et al., 2014). The model is driven by daily temperature, precipitation, and solar radiation and is capable of simulating soil carbon (C), soil water, phosphorus (P), and nitrogen (N) dynamics and their interaction (Keating et al., 2003). Management practices include sowing date, variety selection, irrigation water management, fertilizer application, crop residue management, crop rotations and conservation tillage; this makes the model ideal for assessing the impacts of various management options on resource use. APSIM also allows users to set up atmospheric CO_2_ concentration (Jones et al., 2001), which is ideal for assessing climate change impacts. Furthermore, through the CANOPY module, the model can simulate resource use within intercrop systems. For detailed information on the technical workings of the APSIM model, refer to McCown et al. (1996), Dimes and Revanuru (2004), and Holzworth et al. (2006; 2014).

The CANOPY module determines resources intercepted by each component of the intercrop using leaf area index (LAI), extinction coefficient and height for each crop. Arbitration for water and nitrogen uptake is done based on the module changing the order each day (on a rotational basis) in which the competing species are allowed to capture soil resources. Through the CANOPY module, the model accounts for the vertical profiles of LAI in different species in a mixture (Keating et al., 2003), and assumes a horizontally homogeneous canopy for each species (Gou et al., 2017). The CANOPY module has been published and successfully applied by Smith et al. (2016) and Snapp et al. (2018) for maize and pigeon pea; Carberry et al. (1996) for maize and bean; Chimonyo et al. (2016a) for sorghum and cowpea; and Hoffmann et al. (2020) for various maize intercrop systems. Although Nelson et al. (1998a,b) used APSIM to simulate a maize and *Desmanthus virgatus* intercrop system, the two crops were grown as monocultures and did use the CANOPY module. It was not clear whether Amarasingha et al. (2017) used the CANOPY module when maize and mung bean intercrop systems were simulated in APSIM. In contrast, Knörzer et al. (2011) found that APSIM was unable to simulate wheat-pea and maize-pea intercropping systems in Germany because it strongly underestimates the competitive ability of the species that was planted the first relative to the one that was planted last. In this study, we used the CANOPY module to simulate the effects of climate change on a maize landrace and bambara groundnut intercrop system. The current study, therefore, adds to the existing body of knowledge on the use of ASIM in simulating intercrop systems. It goes further to simulate different management options under different climate change impacts on the intercrop system.

#### Model Calibration, Testing and Application

The calibration and testing of the APSIM were carried out using observed data obtained from field experiments conducted during the 2015/16 growing season for a maize landrace–bambara groundnut intercrop established at the University of KwaZulu-Natal’s Ukulinga Research Farm. Sub-plots comprised intercrop combinations, that is, sole maize landrace, sole bambara groundnut and maize landrace–bambara groundnut intercrop. The irrigated treatments were used for calibration, while the rainfed treatments were used to validate the model. For a detailed description of the experiment, refer to Supplementary Information 1. The simulation files were, therefore, created using observed data collected from the rainfed and irrigated treatment.

#### Met File

For model calibration and testing, a 10-year (2009–2019) weather data file that contained daily estimates of rainfall, minimum and maximum temperatures, solar radiation and reference evapotranspiration was sourced from SASRI weather site (http://sasex.sasa.org.za/irricane/tables/Ash_tables_AR.pl) using the nearest station to the location except for Ukulinga where there is a weather station on-site. With the 5-year climate file, we were able to back-calculate and estimate the initial soil water and initial soil nitrogen at planting. APSIM require an average ambient temperature (TAV) and the annual amplitude in monthly temperature (AMP). These values are calculated using long-term daily minimum and maximum temperatures by software program named “tav_amp.”

#### Soil File

The soil file was generated using soil details at Ukulinga Rresearch Farm. Soils at the research farm have been described as being shallow clayey to clayey loam with medium fertility (Mabhaudhi et al., 2013). The soil file selected to represent this description best was Clay_Shallow_MF_101 mm. The soil module was created using information obtained from Chimonyo et al. (2016b) (Table 1), and this was matched to a pre-existing soil file available in APSIM soil module – Africa (Generic).

#### Crop Files

Within maize APSIM crop file, we used the maize cultivar “mwi_local” as it best described the maize landrace used in terms of days to maturity and yield potential of 3 t ha^−1^. However, slight iterations to genetic coefficients were done using an iterative approach until simulated values were within 9–20% of observed values (Table 2). Since APSIM does not have a bambara groundnut crop file, the groundnut cultivar “kangwana” was modified as it closely resembled bambara groundnut in terms of physiology, growth habit and phenology (Table 2). The groundnut crop module was iterated by first adjusting the reproductive parameters within the crop life cycle (phenology, e.g., time to emergence, first leaf, reproductive stages, and maturity) to resemble what was observed from the monocropped treatment during the field experiment. After that, where simulations disagreed with observations, parameters in the groundnut module were modified in a sequential approach following the order proposed by Boote et al. (2002). The steps were: (1) leaf appearance rate, canopy height, and width, (2) specific leaf area, leaf area index, and partitioning among vegetative organs, including the rate of total biomass accumulation and lastly, (3) onset, rate, and duration of pod addition and seed growth. Besides modifications based on comparisons with the observed data, some parameter modifications were made based on a literature review.

#### Management File

The management file considered planting date, plant densities, fertilizer rate, irrigation and harvest rules. The plant populations used to calibrate and test the model were 2 0 and 2.2 (plants m^−2^) of the maize landrace and bambara groundnut, respectively. The plant population used represented the densities observed in the field experiment and were less than the recommended densities for dryland maize (2.6 plants m^−2^) and bambara groundnut (4.4 plants m^−2^) production (Jensen et al., 2003). Since the field experiment used to calibrate and test the model was conducted in one season, we used the irrigated treatments for calibration and the rainfed treatments to test model. The module “irrigate on the date” was used to apply irrigation on dates corresponding to actual irrigation dates. Observed irrigation applied per event for the field experiment was calculated to be, on average, 15 mm, which was applied thrice during the experiment. Nitrogen fertilizer was applied automatically within 50 cm depth in the soil at a rate of 50 kg ha^−1^ to avoid any nitrogen stress.

### Climate Scenarios

Ukulinga Research Farm is located within quinary sub–catchment 4,697 of quaternary catchment U30J (Schulze et al., 2011). In addition to historical data, the study also used downscaled future climate projections for the Ukulinga quinary. The climate projections were developed by the Council for Scientific and Industrial Research (CSIR) (Table 3) using output from six global climate models (GCMs) from the CMIP5 archive that was forced by Representative Concentration Pathway 8.5 (RCP 8.5). The climates produced under RCP 8.5 were used as they represent the most extreme scenarios. The selection of these six GCMs was based on their ability to provide a reasonable representation of the El Nino-Southern Oscillation (ENSO) phenomenon for the region.

The climate projections were dynamically downscaled to improve spatial resolution to 0.5° (~50 km) using the CCAM regional climate model developed by the Commonwealth Scientific and Industrial Research Organization, CSIRO (McGregor and Dix, 2001, 2008; McGregor, 2005). After that, a multiple-nudging strategy was followed to obtain a downscaling to 0.1° (~10 km) resolution using CCAM in stretched-grid mode over South Africa (see Mabhaudhi et al., 2018a). Climate scenarios were then extracted for the gridded pixel that overlapped quinary sub–catchment 4,697. For application in crop modeling at a local scale, it is necessary to correct for systematic and localized biases in rainfall and temperature projections produced by the climate models. When compared to observed rainfall data from the historical quinary climate database for sub-catchment 4,697, the downscaled climate projections were found to have a substantially larger number of rain days, with many rain days having minimal rainfall depths (i.e., < 0.1 mm). Therefore, a quantile delta mapping method, as described and assessed by Cannon et al. (2015), was applied to bias correct the climate scenarios using a multiplicative factor for rainfall and an additive factor for temperature.

The bias-corrected climate data provide daily rainfall and temperature scenarios for a continuous period from 1961 to 2100. Daily reference crop evaporation (ETo) estimates were then computed as described for the historical data set (see Schulze et al., 2011). Solar radiation for each GCM for Ukulinga was then calculated as described by Schulze and Chapman (2007). The climate database, therefore, satisfied APSIM’s climate file input requirements and was used to develop projections for the past (1961–1991), present (1995–2025), mid-century (2030–2060) and late-century (2065–2095) periods. Throughout the analysis, the “present” timescale was regarded as the baseline.

### Management and Agronomic Scenarios

Two management scenarios were used to develop recommendations for best management practices. The scenarios were as follows.

#### Scenario 1: Planting Dates

Maize production guidelines published by the Department of Agriculture, Forestry and Fisheries suggest that maize should be planted between October 1 and mid-December throughout South Africa [Department of Agriculture Forestry Fisheries (DAFF), 2003]. As it is, South Africa exhibits a wide variation of agro-ecologies, both at the micro and macro level. Due to climate variability and change, this variation has increased, and there is an observed increase in the land area occupied by semi-arid arid agro-ecologies since 2000 (Cairns et al., 2013). Conversely, there is a continual need to redefine planting dates. In this study, we adopted five fixed dates between September 15 to January 15 as this approach is much easier for farmers to use. These dates were assumed to represent early to late planting. However, a significant weakness of this approach is the need to redefine the dates because of continuous shifting in agro-ecologies.

#### Scenario 2: Plant Populations

Model simulations were performed using plant populations that were 50% less to 50% more than the recommended values. Simulations were carried out by maintaining the recommended plant population of one component and changing the other. The total number of simulations was a 3 by 3 factorial with maize populations of 13,000, 26,000, and 39,000 plants ha^−1^ and bambara groundnut populations of 6,500, 13,000, and 19,500 plants ha^−1^. The lower populations would reduce resource competition and improve productivity for either component crop, while higher populations assumed that there was a need to minimize unproductive resource use from the system and improve their productive use. From this, optimum plant populations were determined for both landraces.

### Model Runs

For model calibration and testing, the APSIM intercrop model was run for 10 consecutive years from 2009 to 2019. The 10-year run allowed for soil conditions to stabilize around what was observed in the actual experiment. During the scenario analyses management options were run independently from each other across the six climate projections to minimize the interactive effects of the scenarios. The RCPs were run continuously from 1961–2095 periods.

### Data Analyses

Since APSIM does not calculate WUE directly, simulated outputs of water use (WU in mm) and yield (Y in kg ha^−1^) or biomass (B in kg ha^−1^) were used to determine water use efficiency (WUE in kg mm^−1^ ha^−1^) over the growing season (sowing to harvest) as follows:

(1) $$WUE=\frac{Y/B}{WU}$$

Within the model, WU was determined as crop water uptake from the soil profile by either maize landrace or bambara groundnut crop, i.e., maize Ep and bambara Ep and soil evaporation Es [Ep (for either maize landrace or bambara groundnut +Es)].

For model calibration and validation, model performance was evaluated by comparing simulated (S) vs. observed (O) values for phenology, leaf area index, WU, WUE^B^, grain yield and biomass. Model performance was evaluated using the coefficient of determination (*R*^2^), root mean square error (RMSE) and normalized RMSE (nRMSE). Values of *R*^2^ range between 0 and 1 with high values indicating less error variance. Since the interpretation of *R*^2^ is independent, low values are only acceptable if n is large. However, *R*^2^ values are sensitive to outliers and insensitive to additive, and proportional differences between S and O. The simulation was considered excellent when nRMSE < 10%, good if 10–20%, acceptable or fair if 20–30%, and poor if >30% of the observed mean (Jamieson et al., 1991; Granderson and Price, 2014).

Simulation outputs for yield and water use were subjected to descriptive statistics, *t*-test analyses and generalized linear mixed analysis (GLMM) using R statistical software (version 3.6.0). Descriptive statistics such as means, standard deviations, bubble charts and box and whisker plots were used to analyse outputs. Box and whisker plot can show stability and general distribution of data sets. The GLMM was used to identify significant factors influencing maize landrace and bambara groundnut yield.

### Developing Guidelines

The Food and Agriculture Organization (FAO) suggested a list of guiding questions to review transformative elements within an intervention (Carter et al., 2018). These questions are meant to provide clarity on the adaptation planning process. In this study, we adopted selected questions to assess the implications of the research, provide actionable recommendations and provide a way forward. Key findings were summarized in Table 5 and implications outlined.

## Results and Discussion

### Model Performance

**Figure 1:**
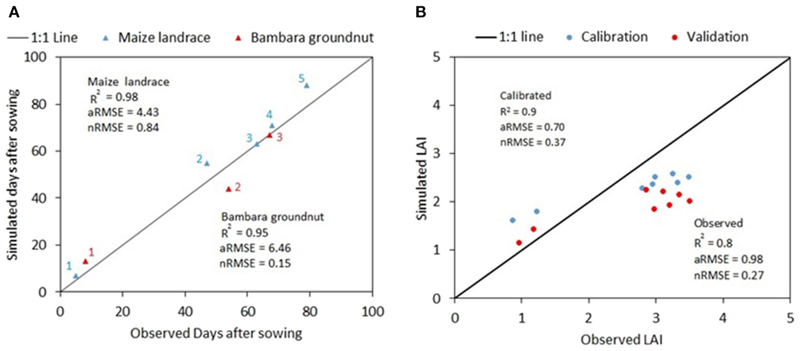
Comparison of observed and simulated (A) phenology (days after sowing) and (B) leaf area index for maize landrace and bambara groundnut during model calibration and validation. Red triangles represent bambara groundnut, and associated numbers 1, 2, and 3 represent phenological stages; emergence, the onset of flowering and start of grain filling respectively. Blue triangles represent maize landrace, and associated numbers 1, 2, 3, 4, and 5 represent phenological stages as emergence, floral initiation, flag leaf formation, the onset of flowering, and start of grain filling respectively.

Comparisons of simulated and observed values for maize landrace and bambara phenology and LAI, and biomass, yield and water use (WU) and water use efficiency (WUE) are given in Figure 1 and Table 4, respectively. For phenology, the close alignment of the points to the 1:1 line indicates that the model was able to simulate the maize landrace and bambara phenology correctly. The model could explain more than 90% of the variation of either crop in phenological stages (Figure 1). During the calibration, the nRMSE for the system LAI was < 10% of the observed LAI for the maize landrace and bambara groundnut intercrop system. The nRMSE for the system LAI during model validation increased slightly to 14%; this implied good simulation for the intercrop system grown under rainfed conditions. Reasonable simulations of crop water use (WU) by the model during calibration, were also observed (RMSE = 41 mm); however, during model validation, WU was over-estimated by 48%. The output suggests that the APSIM model might not be sensitive to water. A closer look at model outputs for maize landrace and bambara groundnut simulated under irrigated (used for calibration) and rainfed (used for validation) conditions showed that transpiration was mostly unaffected by the reduction in water availability. In nature, the low availability of water results in a reduction in transpiration due to the reduction in stomatal conductivity. Field results showed no significant differences between the irrigated and rainfed treatments (see Supplementary Information 1). In this case, the model appropriately captured maize landrace and bambara groundnut physiology.

For biomass and grain yield, the model tended to overestimate the outputs for maize landrace and bambara groundnut. During model calibration, simulated yield and biomass for maize landrace as 11 and 16% higher than observed, and this implied reasonable simulation. Model simulation of maize landrace yield and biomass under rainfed conditions were satisfactory (RSME = 49 and 267 kg ha^−1^) However, simulated yield and biomass for bambara groundnut were 32 and 55% higher than those for observed yields. The performance of APSIM would suggest that, for improved model simulations, additional parameterisation may be required to simulate bambara groundnut adequately. The WUE calculated based on model simulated biomass (WUE^B^) of both the maize landrace and bambara groundnut showed a good fit (1 and 4 kg mm^−1^ ha^−1^, respectively), for simulated and observed results (Table 4). Then again, the bambara cultivar used to calibrate the crop file was a landrace selection. It could be that performance under low water availability had adverse effects on its productivity, and model did not capture this response. Considering that the model was still able to simulate low yields for the maize landrace and bambara groundnut and possible errors in the observation data (e.g., iterated cultivar parameters), the APSIM model performance was considered to be acceptable for the simulation of the intercrop system.

### Change in Climate During the Growing Season

**Figure 2:**
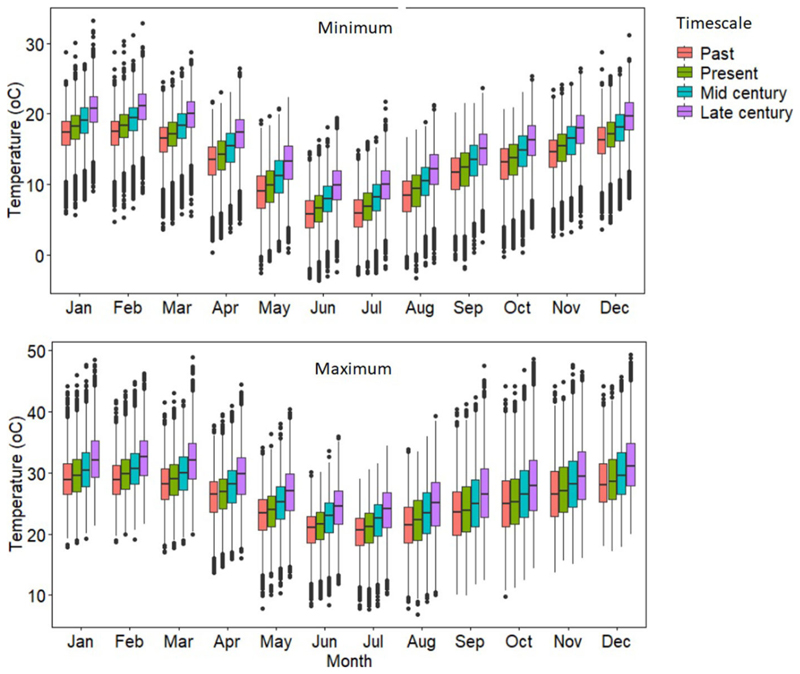
Distribution of average monthly minimum (A) and maximum (B) temperature data for the different timescales (past, present, mid-, and late-century) as simulated by the six GCMs (ACC, CSS, CNR, GFD, NOR, and MPI).

Dynamically downscaled and bias-corrected climate projections for six GCMs forced by RCP 8.5, together with an impact model APSIM 7.7, were used to simulate bambara groundnut and maize landrace yields for past, present, mid-, and late-centuries. The primary aim was to assess how climate change impacts on yield, WU and WUE of the maize landrace and bambara groundnut intercrop. Secondary to that, we assessed the impacts of various management options on mitigating the impacts of climate change. The median value of climate change projections for minimum and maximum temperatures for Ukulinga showed a consistent warming trend across all months from past to late century. Figure 2 indicates a warmer future (mid- and late-century) with mean maximum temperature increasing by 4.5°C relative to the baseline maximum temperature of 24°C. This suggests an increased probability of heat stress, especially for the maize landrace. This warming trend across the selected timescales is consistent with projected trends for South Africa (Mangani et al., 2018).

The six GCMs project an increase in mean minimum temperatures in the future (mid- and late-century) that ranges from 2.0–4.8°C from a minimum baseline temperature of 13°C. The projected increases suggest an increased probability of hot nights and longer and more frequent heatwaves. The warmer temperatures may result in a faster accumulation of heat units and a reduction in growth duration and accumulation of photosynthesis and increase in night-time respiration, all resulting in reduced crop yield (Schlenker and Roberts, 2009). Unlike bambara groundnut (C3 plant), maize (C4 plant) generally originates from warmer climates (Leff et al., 2004; Jia et al., 2016) and thus, may be more resilient to projected increases of temperature (Choudhary et al., 2019). Then again, for bambara groundnut, optimum temperatures range between 28 and 35°C and the lethal temperature has been reported to be 50°C (Soni et al., 2015). The wide temperature adaptation makes the crop ideally suited for building resilience to cropping systems located in areas where temperature increases have been projected.

**Figure 3:**
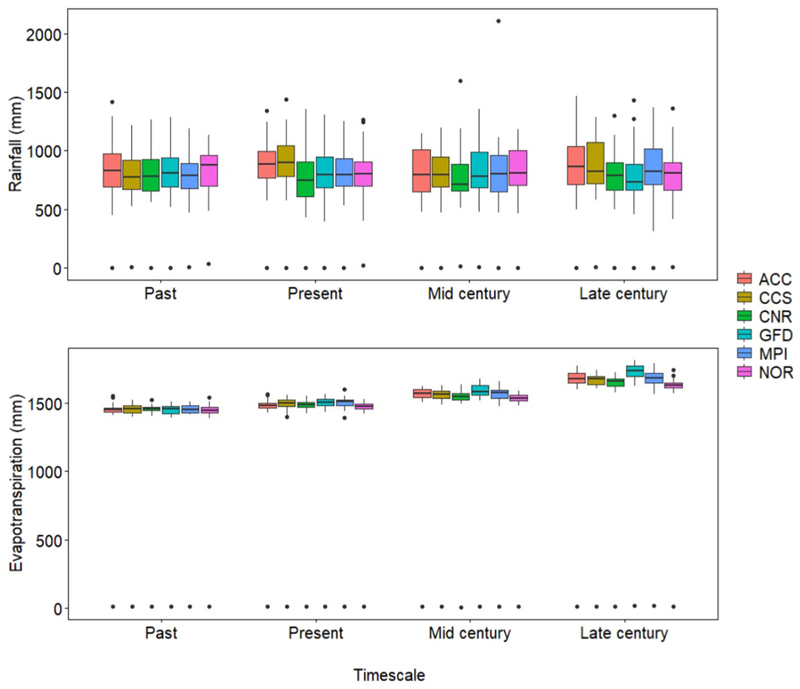
Rainfall data representative of four different timescales (past, present, mid-, and late-century) as simulated by the six GCMs (ACC, CSS, CNR, GFD, NOR, and MPI). The average yearly rainfall calculated from observed rainfall data received between 2004 and 2019 was used as the mean annual rainfall.

Results across the GCMs show that mean annual rainfall (MAP) for the future is projected to remain somewhat unchanged (Figure 3). for the late-century period, data showed that ACC and CCS predict a 10.6 and 8.3% increase in MAP, respectively, while slight reductions of 3.5 and 2.5% are predicted by CNR and NOR respectively. However, the more extended box and whisper plots for ACC predict an increase in the inter-annual variability of mean rainfall (750 mm) (Figure 3). This suggests an increase in the probability of extreme weather events such as drought and floods. In all instances, projected ET_o_ was observed to be higher (35%) than projected rainfall and is set to increase in the future (mid and late century) (Figure 3). In this regard, the rainfall: ET_o_ ratio is projected to decrease in the near future. The increase in ET_o_ is consistent with the projected increase in minimum and maximum temperature and suggests an increase in crop water stress (Zhao et al., 2017). Then again, intercrop systems with cereals and legumes are advantageous as the cereal over-story can lower canopy temperature and minimize evaporative losses (Eskandari, 2011; Chimonyo et al., 2016b). The modification of microclimate within intercrop systems makes it an ideal system to mitigate against projected temperature and ET_0_ increases. Our results suggest that, while the tolerances of traditional crops to high temperatures may vary, intercropping crop species with different physiological and morphological traits can be a strategy to increase the resilience of marginalized production systems to projected temperature ET_0_ increases.

### Yield, Water Use and Water Use Efficiency

**Figure 4:**
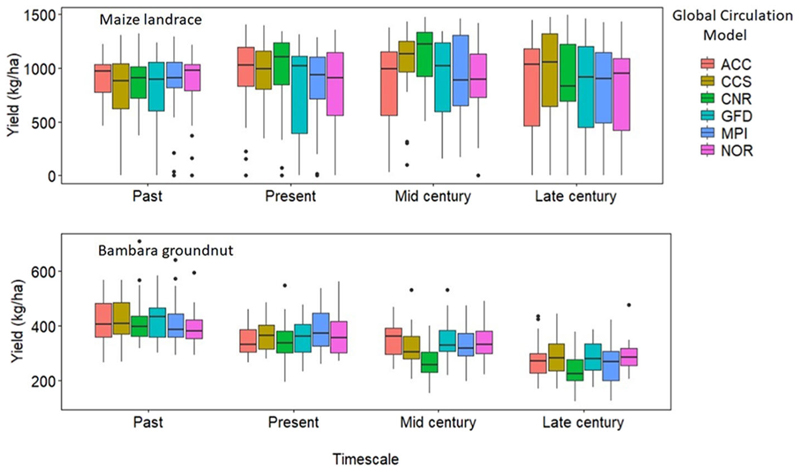
Simulated yield (kg ha^−1^) for maize landrace and bambara groundnut during four different timescales (Past, Present, Mid-, and Late-century) under rainfed conditions obtained from the six GCMs (ACC, CSS, CNR, GFD, NOR, and MPI). The effect of timescale on maize landrace grain yield - *F*-statistic: 3.492 on 3 DF, *P* = 0.01. The effect of the interaction between GCM and timescale on bambara groundnut grain yield - *F*-statistic: 1.953 on 15 DF, *P* = 0.01.

Across the GCMs, yield trends for intercropped bambara groundnut showed a gradual reduction toward the late century by 24% when compared to the baseline yield of 365 kg ha^−1^. The observed trend for simulated bambara groundnut yield was late-century (285 ± 57) < mid-century (323 ± 62) < present (365 ± 67) < past (450 ± 65 kg ha^−1^) (Figure 4). Across the GCMs, the magnitude of change in simulated bambara groundnut yield during the mid- and late-century periods was consistent with corresponding projected increases in ET_o_ and temperature. On the other hand, the mean yield trends for intercropped maize landrace across the GCMs and time scales were inconsistent with projected increases in ET_o_ and temperature. The observed trend for simulated maize landrace yield was past (845) < late-century (855) < present (923) < mid-century (967 kg ha^−1^). For mid-century, although the model predicted a slight increase in maize landrace yield, results also showed larger yield variations relative to past and present. Standard deviations for the intercropped maize landrace yield were past (288) < present (363) < mid-century (351) < late-century (436 kg ha^−1^) (Figure 4). These results are in line with the increased probability of extreme weather events such as drought and floods (Schulze, 2011). Although bambara groundnut yield decreased across the time scales, the magnitude of yield variations within each timescale and GCM was somewhat consistent with an average standard deviation of 63 kg ha^−1^. Within each timescale, our results would suggest that yields of bambara groundnut are more stable to climate fluctuation; however, it could be more sensitive to significant climate changes.

**FIGURE 5:**
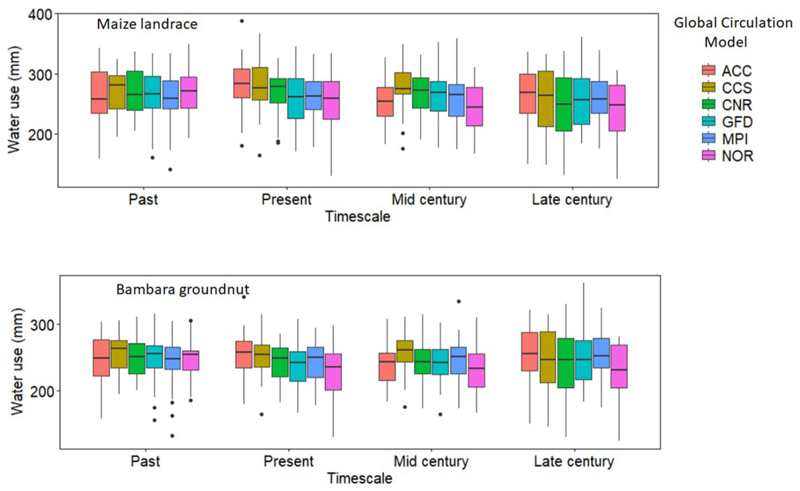
Calculated water use (mm) of maize landrace and bambara groundnuts from soil evaporation (Es), crop water use (Ep) as simulated by APSIM across the six GCMs (ACC, CSS, CNR, GFD, NOR, and MPI) for each timescale (past, present, mid-century, and late-century). The effect of timescale on maize landrace water use - *F*-statistic: 2.989 on 3 DF, *P* = 0.03. The effect of GCM on maize landrace water use - *F*-statistic: 2.6392 on 5 DF, *P* = 0.02. The effect of GCM on bambara groundnut water use - *F*-statistic: 3.315 on 5 DF, *P* = 0.005.

**FIGURE 6:**
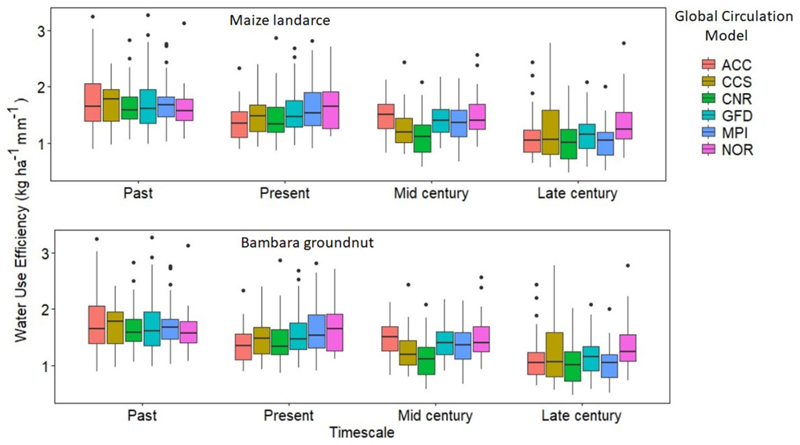
Calculated water use efficiency (kg ha^−1^ mm^−1^) for maize landrace and bambara groundnut across the six GCMs (ACC, CSS, CNR, GFD, NOR, and MPI) for each timescale (Past, Present, Mid-, and Late-century). Bambara groundnut *F*-statistic: 2.122 on 3 DF, *P*-value: 0.1 and bambara groundnut *F*-statistic: 4.543 on 3, *P* = 0.003.

The trend for water use in the intercropped maize landrace and bambara groundnut was inconsistent across the GCM and timescales. Overall, CCS predicted the highest water use (265 and 253 mm), while the lowest was under NOR (242 and 235 mm) for maize landrace and bambara groundnut, respectively (Figure 5). Differences in simulated WU across the GCMs could be that each climate model has been developed based on its assumptions and unique mathematical representations of physical climate system processes, providing different climate projections (Confalonieri et al., 2016). There were slight reductions in WU across the timescale; however, based on the pairwise *t-*test analysis, the reductions were not significant (*P* > 0.05). On the other hand, simulated results for crop water use efficiency (WUE) for intercropped bambara groundnut showed a reduction across time scales. The trend was such that past (1.78 ± 0.45) > present (1.52 ± 0.41) > mid-century (1.37 ± 0.38) > late century (1.16 ± 0.42 kg ha^−1^ mm ^1^) (Figure 6). The observed trend was consistent with the observed reduction in future yield. Large inconsistencies were observed for maize landrace WUE across GCMs and time scales. For example, CNR predicted the highest water use (4.01 ± 1.98 kg ha^−1^ mm^−1^), while the lowest was under NOR (3.25 ± 1.05 kg ha^−1^ mm^−1^). The trend for maize landrace WUE across the timescale was such that present (3.58 ± 1.25) < past (3.62 ± 0.81) < late century (4.37 ± 1.38) < mid-century (4.56 ± 1.82 kg ha^−1^ mm^−1^) (Figure 6). The observed trend was consistent with the simulated improvements of maize yield.

### Optimizing the Performance of Bambara Groundnut in Intercrop Systems

#### Impacts of Planting Density on Yield and Water Use Efficiency of a Maize Landrace and Bambara Groundnut Intercrop System

**FIGURE 7:**
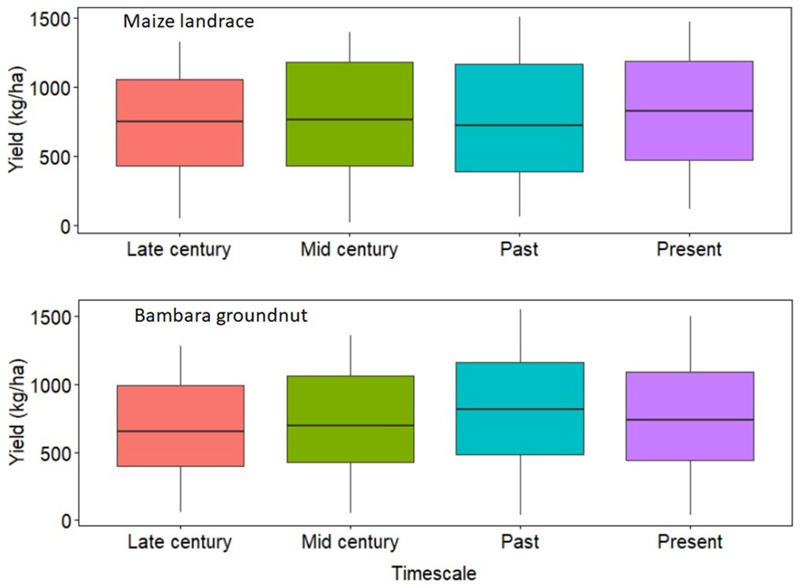
Simulated maize landrace and bambara groundnut yields (kg ha^−1^) across different timescales from six GCM (ACC, CSS, CNR, GFD, NOR, and MPI). The effect of timescale on bambara groundnut yield *F*-statistic: 5.031 on 3 DF, *P* = 0.001.

**FIGURE 8:**
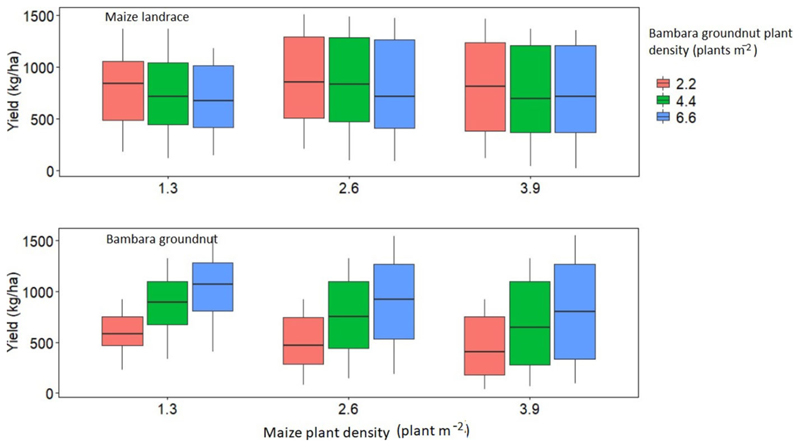
Simulated yield response of maize landrace and bambara groundnut to plant population (plant m^−2^) for climate scenarios obtained from six GCM (ACC, CSS, CNR, GFD, NOR, and MPI). Different colors of boxplots represent the bambara groundnut plant density (plant m^−2^). The effect of the interaction between maize landrace plant density and bambara groundnut plant density on maize landrace grain yield - *F*-statistic: 62.47 on 8 and 891 DF, *P* = 0.000. The effect of the interaction between maize landrace plant density and bambara groundnut plant density on bambara groundnut grain yield - *F*-statistic: 38.93 on 24 and 875 DF, *P* = 0.000.

Simulation results of yield and WU for intercropped maize landrace and bambara groundnut across the six GCM were not significantly (P > 0.05) different; therefore, the results presented in this section are average values across the six GCMs. Across timescales, the trend for maize landrace yield was past (850 ± 288) < late-century (853 ± 443) < present (893 ± 359) < mid-century (959 ± 362 kg ha^−1^) (Figure 7). Increasing maize landrace plant population resulted in a significant increase in mean yield but did not affect WUE (Figure 8). Regardless of bambara groundnut plant population, increasing maize landrace plant population resulted in a 12% reduction in its mean yield while reducing the population resulted in an 8% improvement in its mean yield (Figure 8). On the other hand, simulated yield and WUE for intercropped bambara groundnut was significantly (*P* < 0.05) affected by timescales and by the interaction between maize landrace and bambara groundnut planting date.

**FIGURE 9:**
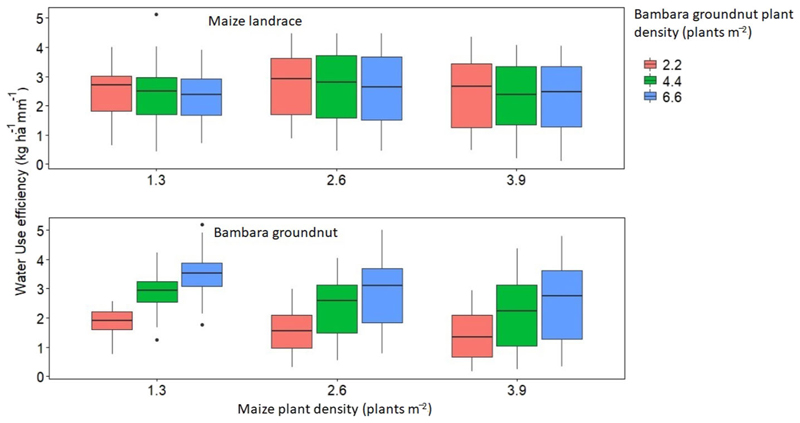
Calculated water use efficiency maize landrace and bambara groundnut plant population (plant m^−2^) for climate scenarios obtained from six GCM (ACC, CSS, CNR, GFD, NOR, and MPI). The colored boxplots represent the bambara groundnut plant density (plant m^−2^). The effect of maize landrace plant density on maize landrace WUE - *F*-statistic: 6.78 on 2 and 891 DF, *P* = 0.001. The effect of the interaction between maize landrace plant density and bambara groundnut plant density on bambara groundnut WUE - *F*-statistic: 38.93 on 24 and 875 DF, *P* = 0.000.

Across timescales, the trend for bambara groundnut yield was past (806 ± 406) > present (760 ± 404) > mid-century (717 ± 359) > late-century (674 ± 332 kg ha^−1^) (Figure 7). Likewise, the trend for bambara groundnut WUE was past (2.54 ±1.10) > present (2.37 ± 1.23) > mid-century (2.33 ± 0.98) > late-century (2.17 ± 0.89 kg ha^−1^ mm^−1^) (Figure 8). Although the observed WUE for bambara groundnut in the late century represented an 87% improvement relative to the baseline (1.16 ± 0.42 kg ha^−1^ mm^−1^) for the same period, there was a 52% increase in its variability. Increasing bambara groundnut plant population increased simulated yield by 43% at the highest plant population, but also increased yield variability (standard deviation). The simulated mean yields (in kg ha^−1^) and corresponding standard deviations were 520 ± 247 < 777 ± 353 < 921 ± 406 for intercropped bambara groundnut simulated at 2.2, 4.4 and 6.6 plants m^−2^, respectively (Figure 9). A similar trend was observed for the calculated WUE (in kg ha^−1^ mm^−1^), which was 1.61 ± 0.60 < 2.41 ± 0.94 < 2.98 ± 1.07 for intercropped bambara groundnut at 2.2, 4.4 and 6.6 plants m^−2^ (Figure 9). This would suggest that the currently recommended plant populations of 4.4 plants m^2^ might be low for optimum use of resources such as water.

There was a reduction in simulated mean yield for bambara groundnut with the increase in maize landrace plant population. The trend for bambara groundnut yield was 841 ± 305 > 720 ± 379 > 657 ± 422 (kg ha^−1^) when intercropped with maize landrace at plant populations of 1.3, 2.6 and 3.9 plants m^−2^, respectively (Figure 7). Similar to the simulated yield trend of intercropped bambara, increasing maize plant population resulted in a reduction of calculated bambara WUE and an increase in its variability (standard deviation) (Figure 7). The reduction of simulated yield and WUE maxima and minima for bambara groundnut and the increase in yield variability under high maize landrace plant populations could be attributed to increased competition for resources with the maize landrace. Peake et al. (2008) observed that increasing maize plant populations beyond a specific limit could increase the risk of crop failure due to an increase in competition for water and solar radiation. In cases were both yields of the maize landrace and bambara groundnut are desired by a farmer, it might be worthwhile to reduce maize landrace plant populations to maximize yield for bambara groundnut. Alternatively, there is a need to improve water availability through rainwater harvesting and conservation techniques to reduce competition for water within the intercrop.

#### Impacts of Planting Dates on Yield and Water Use Efficiency of Maize Landrace and Bambara Groundnut Intercrop System

**FIGURE 10:**
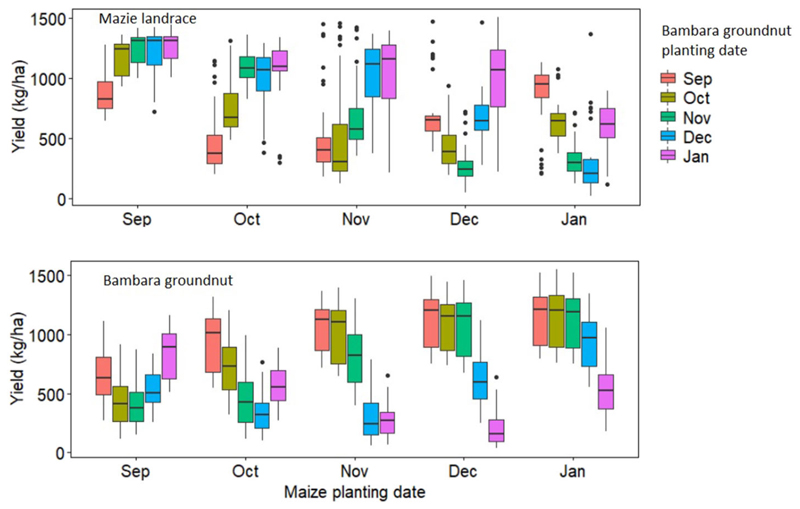
Simulated yield for maize landrace and bambara groundnut across different planting date combinations under rainfed conditions obtained from six GCMs (ACC, CSS, CNR, GFD, NOR, and MPI). The x-axis represents the maize landrace planting dates and the colored boxplots represent the planting date for bambara groundnut. The effect of the interaction between maize landrace planting date and bambara groundnut planting date on maize landrace grain yield - *F*-statistic: 49.93 on 24 and 875 DF, *P* = 0.000; The effect of the interaction between maize landrace planting date and bambara groundnut planting date on bambara groundnut grain yield - *F*-statistic: 75.37 on 24 and 875 DF, *P* = 0.000.

**FIGURE 11:**
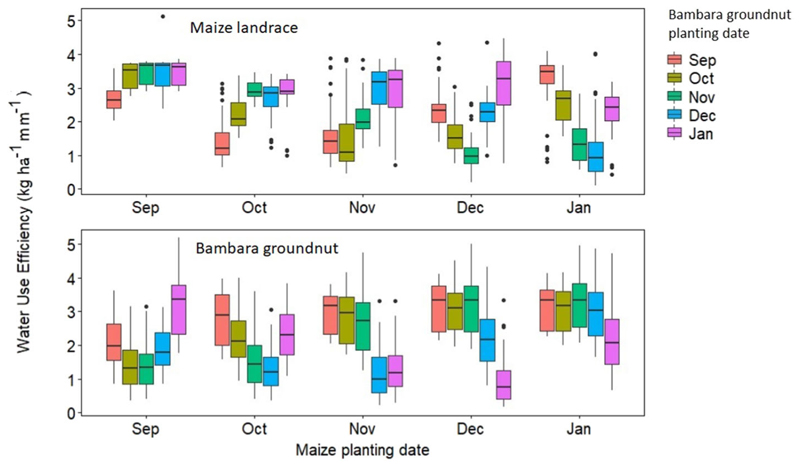
Calculated water use efficiency (kg ha^−1^ mm^−1^) for maize and bambara groundnut across different planting date combinations under rainfed conditions obtained from six GCMs (ACC, CSS, CNR, GFD, NOR, and MPI). The x-axis represents the maize landrace planting dates and the colored boxplots represent the planting date for bambara groundnut. The effect of the interaction between maize landrace planting date and bambara groundnut planting date on maize landrace WUE - *F*-statistic: 38.93 on 24 and 875 DF, *P* = 0.000 and The effect of the interaction between maize landrace planting date and bambara groundnut planting date on maize landrace WUE - *F*-statistic: 35.16 on 24 and 875 DF, *P* = 0.000.

Simulation results for yield and WU for maize landrace and bambara groundnut across the six GCM were also not significantly different (*P* > 0.05); therefore, the results presented in this section were average values across the six GCMs. Simulated yield for maize landrace and bambara groundnut was significantly (*P* < 0.05) affected by the interaction of their planting dates (Figures 10, 11). Overall, early planting (September) of maize landrace or bambara groundnut resulted in higher simulated yields relative to late planting (January). Across the planting dates, mean yield trends for intercropped bambara groundnut was September (992 ± 296) > October (889 ± 357) > November (681 ± 383) > December (548 ± 301) >January (486 ± 283 kg ha^−1^). On the other hand, calculated WUE trend for intercropped bambara groundnut was September (2.81 ± 0.78) > October (2.53 ± 0.96) > November (2.46 ± 1.15) > January (1.99 ± 1.12) > December (1.94 ± 1.01 kg mm^−1^). For maize landrace, mean yield trends was September (1,052 ± 116) > October (902 ± 197) > November (835 ± 213) > December (648 ± 261) >January (596 ± 283 kg ha^−1^). On the other hand, calculated WUE trends for intercropped bambara groundnut was September (3.01 ± 0.88) > October (2.73 ± 0.76) > November (2.33 ± 0.95) > January (2.09 ± 1.00) > December (1.84 ± 0.66 kg mm^−1^). The calculated WUE was lower (24–107%) than the calculated WUE baseline of 4.12 kg mm ^1^. This could be attributed to a reduction in the lower quartile values (Figure 10), which would suggest an increase in maize landrace yield gap with later planting. According to several research outputs, climate change is expected to reduce the length of the growing season and increase the occurrence of dry spells (Mitchell et al., 2015; Ajetomobi, 2016; Paff and Asseng, 2018). Despite the loss of growing days, our result suggests that, when planting on the same day, early planting (September) may ensure stable yields and WUE are obtained. Rezvani Moghaddam et al. (2014) found that early planting could be used as an adaptation strategy for maize under future climate in arid regions of Iran. Hussain et al. (2018) also highlight that, regardless of planting date, yield responses are highly dependent on resource availability and distribution, in this case, rainfall.

Intercropping bambara ground at different planting dates to maize landrace improved the mean yield (Figure 10), and WUE (Figure 11) provided it was done before November 15. Planting bambara groundnut a month earlier than maize landrace - for instance, planting in the former September and maize landrace in October, resulted in a 176 and 57% increase in its mean yield and WUE, respectively, relative to the baselines. Planting bambara groundnut 2 and 3 months earlier than maize landrace resulted in a 184% increase in yield (Figure 10) and improved WUE by 61% increase in WUE (Figure 11). Planting maize landrace a month earlier than bambara groundnut - for instance, planting in September and bambara groundnut in October, resulted in the most significant mean yield increase (56%) relative to the baseline (Figure 11). The asynchronous or sequential planting did not result in the overlap in critical phenological stages for both the maize landrace and bambara groundnut. This minimized the competition for water and other resources and maximized resource use through extending canopy duration, therefore improving yield and WUE for maize landrace and bambara groundnut within the intercrop. When critical periods overlap, Yu et al. (2016)suggested that the competitive balance in cereal-legume intercrops can be maintained by planting the legumes earlier than the cereals. This can be viewed as a strategy to minimize the risk of yield loss in the event of intermittent dry spells within the season. However, sequential cropping in rainfed systems is constrained by the length of growing period (Inthavong et al., 2011; Kotir, 2011; Vadez et al., 2012; Duku et al., 2018; Minda et al., 2018). In this study, we did not assess the impacts of climate change on changes in the length and shifts of the growing season, nor the probability of dry spell occurrence and duration.

## Way Forward and Recommendations

Overall, crop simulation models (CSM) and climate scenarios provided a monitoring and surveillance system to identify climate trends and associated impacts on intercropped maize landrace and bambara groundnut yield and WUE. In this regard, the use of a CSM driven by climate projections from six GCMs provided an opportunity to assess the suitability and sustainability of intercropping traditional crops as a potential climate adaptation strategy under low input–low output production systems. Our study demonstrated that the availability of a range of GCM outputs provided useful indications of the potential magnitude of yield and WUE changes and the temporal variation that could occur for the intercrop system. This type of analysis was, therefore, helpful in improving our understanding of the type of climate risk on the maize landrace and bambara groundnut intercrop system (Table 5). We recommend that the use of a CSM with GCM output should be considered when assessing the applicability of agricultural adaptation strategy.

Our results further showed that, at present, functional crop diversity could enhance crop productivity, stability, and thus food security, through efficient water utilization. Also, the adoption of asynchronous or sequential planting and moderating plant populations of either maize landrace or bambara groundnut can be viewed as a low-cost option to improve productivity and WUE under increasing temperature. This allows for the identification of short, medium and long-term strategies to aid in mitigating the impacts of climate change on the productivity and WUE of maize landrace and bambara groundnut intercrop system (Table 5). However, these approaches do not represent the diversity and breadth of adaptation strategies that can be adopted by marginal farmers.

To better represent adaptation, there is a need to expand the research to consider other management strategies (e.g., other traditional crop species, different cropping sequences, fertilization, rainwater harvesting and soil conservation techniques) (Seyoum et al., 2017). In addition, more system (agroecosystem) and place-based approaches that can represent local context, knowledge and aspects of food and nutrition security other than availability (e.g., nutrition, access, utilization and stability) may be required (Beveridge et al., 2018). To increase the contribution of agriculture to improving food and nutrition security, poverty reduction, and enhance rural economic development, climate impact modeling studies should be coupled with social, economic and environmental system models. This will ensure that traditional crops and associated cropping systems are assessed in a holistic manner that informs their sustainable integration into existing cropping systems. However, the adoption of traditional crops and intercropping should not be viewed as a panacea to solve all climate adaptation challenges, nor is it the only adaptation strategy. The inclusion of traditional crops into cropping systems should be considered as a complementary strategy to increasing climate resilience in marginal cropping systems.

A gap between the potential and practical realization of adaptation exists, and the evidence from our study supports the view that adaptation strategies need to be both climate-informed and context-specific to be viable (Beveridge et al., 2018; Carter et al., 2018). The cultivation of traditional crops has been done for millennia; however, to our knowledge, no study has quantified the yield and WUE responses in an intercrop system and under the impacts of climate change. Further to this, the FAO guidelines and key questions provided a useful framework to contextualize the observed results in an informative manner (Table 5) and less prescriptive. With the impacts of climate variability and change, our results provide evidence that adapting agronomic management could allow for sustainable intensification of the traditional systems through improved resource use efficiencies. However, we acknowledge that this type of study should be repeated across other agro-ecologies different from that of Ukulinga, allowing for more robust crop management practices and adaptation strategies to be identified.

The calibration and validation process concerning the APSIM maize landrace and bambara groundnut intercrop study was the first attempt to evaluate the impacts of climate change on growth and water use. In this study, data to calibrate and validate the model were obtained from irrigated and rainfed experiments, respectively, in the same growing season. This may not fully meet strict requirements for using an independent data set for model validation. Therefore, future studies should repeat the experiment across various agro-ecologies and time scales different from that of the calibration data set; this will allow for better validation of model performance and robustness.

## Conclusions

There is a high probability that yield and WUE for intercropped bambara groundnut will decrease in the near to distant future if current management options are maintained. Assuming future rainfall remains mostly unchanged, the primary limitations to intercropped bambara groundnut yield and WUE will be temperature and ET_o_ under minimal rainfall changes. However, projected changes in temperature and ET_o_ will increase yield and WUE variability for a maize landrace and bambara groundnut intercrop system. Improving WU, through increased plant density or asynchronous planting of the maize landrace and bambara groundnut mitigated the negative impacts of climate change on yield and WUE. In this regard, optimum plant management can optimize traditional production systems. Thus, intercrop systems of maize landrace and bambara groundnut should be promoted as a potential future system for climate change adaptation in rainfed production systems.

While the results of these simulations are limited to one agro-ecology and a single intercrop system, the findings confirm the views that several traditional crops are drought tolerant and thus, are suitable for cultivation in marginal agricultural production areas. Furthermore, intercropping them can increase system resilience under climate change. The concept of WUE, among other parameters, has been suggested in selecting management options that can sustainably increase productivity under climate changes, heat and water stress, and interactions among them.

Intercropping maize landraces and bambara groundnut with the appropriate place-based management practices can be used as an adaptation strategy in environments that are projected to face increasing water scarcity. Reduced land and water demand from intercropping maize landraces and bambara groundnut and improved water use efficiencies mitigate the risks associated with increasing climate variability and extreme events such as drought. For resource-poor farmers that are inherently risk-averse, the production of traditional crops such as maize landraces and bambara groundnut, and their optimisation through inexpensive management strategies present an opportunity to build resilient cropping systems. Our results have important implications on how traditional crops and cropping systems should be viewed, in that their incorporation into marginal production systems can be an alternative adaptation strategy that may lead to sustainable intensification outcomes under increasing climate risk.

## Supplementary Material

Supplementary material

## Figures and Tables

**TABLE 1:** Soil water properties at different depths for soil at the experimental site.

Texture	BD^a^		HC^b^	PWP^c^	FC^d^	TAW^e^	SAT^f^		${\text{K}}_{\text{SAT}}^{\text{g}}$
	gm^−3^				mm m^−1^				mm h^−1^
Clay	1.35		0.33	294	416	152	489		19,70

a *Bulk density;*

b *Hygroscopic moisture content;*

c *Permanent wilting point;*

d *Field capacity;*

e *Total available water;*

f *Saturation;*

g *Hydraulic conductivity*.

**Table 2:** Modification of groundnut crop coefficients based on experimental data and data obtained from the literature.

Parameter	Description	Default peanut crop file (*cv* kangwana)	New bambara groundnut crop file
Temp units	Temperature table for thermal time	9.0 29.0 39.0	8.5 28.0 38.0
leaf_dm_init	Initial leaf dry matter	0.045	0.035^b^
ratio_root_shoot	ratio_root_shoot	0 0 1.0 1.0 0.33 0.33 0.087 0 0 0 0	0 0 1.0 0.67^a^ 0.33 0.33 0.087 0 0 0 0
frac_leaf units	Fraction of remaining dry matter allocated to leaves	0 0 0.58 0.58 0.58 0.45 0.45 0 0 0 0	0 0 0.60^b^ 0.60^b^ 0.60^b^ 0.55^b^ 0.55^b^ 0 0 0 0
frac_pod units	Fraction of dry matter allocated to pod or multiplier of grain dry matter to account for pod dry matter	0 0 0 0 0 0.18 0.25 0 0 0 0	0 0 0 0 0 0.30^b^ 0.45^b^ 0 0 0 0
leaf_size	leaf_size	2,000 4,000 4,000 4,000 4,000	4,800 4,800 4,800 4,800 4,800^b^
sla_max description	Maximum specific leaf area for delta LAI	35,000 30,000 25,000 20,000 20,000 20,000 20,000	45,000 45,000 40,000 40,000 38,000 34,000 30,000
hi_incr	Rate of HI increase	0.0056	0.0024
hi_max_pot	Maximum harvest index potential	0.45	0.35^a,c^
Floral initiation (°Cd)		680	220^a^
Flowering (°Cd)	Time from flowering to start grain fill	300	340^a^
Start_grain_fill	Duration of grain filling	440	550^a^
End_grain_fill	Duration of seed maturation	10	85*^a^*
Height (mm)	Plant height	700	400^a^
		**Default maize crop file (*cv*** **mwi_local)**	**Iterated maize crop file**
tt_flower_to_maturity description (°Cd)	Time from flowering to maturity	780	750
PotKernelWt (g 100 kernels^−1^)	Potential kernel weight	260	160

a *Field observation;*

b *model iteration;*

c Karunaratne et al. (2010).

**Table 3:** Global climate models used in this study.

Abbreviation	Model name	Model center	Horizontal resolution
ACC	ACCESS1-0	Commonwealth Scientific and Industrial Research Organization, Australia (CSIRO), and Bureau of Meteorology, Australia (BOM)	1.250 × 1.875°
CCS	CCSM4	National Center for Atmospheric Research (NCAR), USA	0.9424 × 1.250°
CNR	CNRM-CM5	Center National de Recherches Meteorologiques, Meteo-France, France	1.4005 × 1.4065°
NOR	NorESM1-M	NorESM (Norwegian Earth System)	1.250 × 0.940°
GFD	GFDL-CM3	Geophysical Fluid Dynamics Laboratory, USA	2.000 × 2.500°
MPI	MPI-ESM-LR	Max Planck Institute for Meteorology, Hamburg Germany	1.8653 × 1.875°

**Table 4:** Calibration and validation results for observed and simulated outputs for maize landrace and bambara groundnut intercrop system for final biomass (kg ha^−1^), yield (kg ha^−1^) and intercrop system water use (mm).

		Observed	Simulated	RSME
**Model calibration (Irrigated treatment)**				
Maize landrace (kg ha^−1^)	Yield	820	918	98
	Biomass	2,370	2,741	371
Bambara groundnut (kg ha^−1^)	Yield	230	244	14
	Biomass	1,060	1,375	315
Intercrop WU (mm)		291	332	41
Intercrop WUE^b^ (kg mm^−1^ ha^−1^)		11	12	1
**Model validation (rainfed treatment)**				
Maize landrace (kg ha^−1^)	Yield	870	919	49
	Biomass	2,470	2,737	267
Bambara groundnut (kg ha^−1^)	Yield	150	213	63
	Biomass	950	1,248	398
Intercrop WU (mm)		179	266	87
Intercrop WUE^b^ (kg mm^−1^ ha^−^^1^)		19	15	4

**Table 5:** FAO guidelines and key questions for assessing the impacts of adaptation strategy.

FAO Guideline Question	Key Findings	Comments	Implication
How can CSMs and climate scenarios assist in articulating decision windows?	They provided a monitoring and surveillance system to identify short-, medium- and long-term climate trends and associated impacts on intercropped maize landrace and bambara groundnut yield and WUE Data and trends on climate indicators allowed for the identification of possible responses to increasing system resilience	By late century, there will be an increase in temperature and ETo, while rainfall remains somewhat unchanged Maize landrace yield responses are in line with rainfall trends Bambara groundnut yield and WUE will be negatively impacted by increasing temperature Adopting “better bet” management options in bambara can mitigate the projected impacts of climate change and improve the overall performance of the intercrop system	Useful for improving understanding of climate risk and impacts Useful in building the resilience of smallholder farming systems to possible impacts of climate change For low input–low output systems, the adoption of traditional crops has the potential to support positive transformative adaptation to climate change
What are the likely short-, medium-, and long-term climate change impacts and risks for agriculture? How does risk shift further into the future?	*Short-term:* an increase in yield variability resulting in increases in yield gaps *Medium-term:* increases in climate risk will increase competition for resources within the intercrop system *Long-term:* reduction in water availability through increases in temperature and evaporative demand	*Short-term:* Use of adaptable crop species and cropping systems can reduce yield minima in marginal systems *Medium-term:* reducing competition of resources within intercrop through enhanced niche differentiation *Long-term*: There is a need to reduce the unproductive loss of water	*Short-term:* intercropping maize landrace and bambara groundnut under recommended guidelines will improve overall system productivity and WUE relative to corresponding monocrop systems *Medium-term:* adopt asynchronous or sequential planting to reduce competition within the intercrop systems *Long-term:* adopt rainwater harvesting and soil water conservation strategies to enhance soil water capture, storage and minimize unproductive loss of soil water
Which of these interventions are likely to stand the test of time rather than becoming obsolete?	Intercropping maize landrace at low plant population and bambara groundnut at high population can sustainably improve yield and WUE of the system under projected climate change Early planting improves yield and WUE of maize landrace and bambara groundnut intercrop system under projected climate change Planting bambara groundnut 2 months earlier than maize landrace can minimize resource competition and enhance productivity	Manipulating planting densities and dates can aid in maintaining the competitive balance within an intercrop system	Sequential cropping in rainfed systems may be constrained by the length of the growing period Good agronomy can result in high yield and WUE

## Data Availability

The raw data supporting the conclusions of this article will be made available by the authors, without undue reservation.

## References

[R1] Abegaz KH (2018). Prevalence of undernourishment: trend and contribution of East African countries to sub-Saharan Africa from 1991 to 2015. Agric Food Secur.

[R2] Ajetomobi JO (2016). Effects of weather extremes on crop yields in Nigeria. African J Food Agric Nutr Dev.

[R3] Amarasingha RPRK, Suriyagoda LDB, Marambe B, Rathnayake WMUK, Gaydon DS, Galagedara LW (2017). Improving water productivity in moisture-limited rice-based cropping systems through incorporation of maize and mungbean: a modelling approach. Agric Water Manag.

[R4] Badu-Apraku B, Fakorede MAB, Badu-Apraku B, Fakorede MAB (2017). Advances in Genetic Enhancement of Early and Extra-Early Maize for Sub-Saharan Africa.

[R5] Beveridge L, Whitfield S, Challinor A (2018). Crop modelling: towards locally relevant and climate-informed adaptation. Clim Change.

[R6] Boote KJ, Mínguez MI, Sau F (2002). Adapting the CROPGRO legume model to simulate growth of faba bean. Agron J.

[R7] Cairns JE, Hellin J, Sonder K, Araus JL, MacRobert JF, Thierfelder C (2013). Adapting maize production to climate change in sub-Saharan Africa. Food Secur.

[R8] Cannon AJ, Sobie SR, Murdock TQ (2015). Bias correction of GCM precipitation by quantile mapping: how well do methods preserve changes in quantiles and extremes?. J Clim.

[R9] Carberry PS, Adiku SGK, McCown RL, Keating BA, Ito O, Johansen C, Adu-Gyamfi JJ, Katayama K, Kumar Rao JVDK, Rego TJ (1996). Dynamics of Roots and Nitrogen in Cropping Systems of the Semi-Arid Tropics.

[R10] Carter R, Ferdinand T, Chan C (2018). Transforming Agriculture for Climate Resilience: A Framework for Systemic Change.

[R11] Chibarabada TP, Modi AT, Mabhaudhi T (2015). Bambara groundnut (Vigna subterranea) seed quality in response to water stress on maternal plants. Acta Agric Scand Sect B — Soil Plant Sci.

[R12] Chibarabada TP, Modi AT, Mabhaudhi T (2017). Nutrient content and nutritional water productivity of selected grain legumes in response to production environment. Int J Environ Res Public Health.

[R13] Chimonyo VGP, Modi AT, Mabhaudhi T (2016a). Simulating yield and water use of a sorghum–cowpea intercrop using APSIM. Agric Water Manag.

[R14] Chimonyo VGP, Modi AT, Mabhaudhi T (2016b). Water use and productivity of a sorghum-cowpea-bottle gourd intercrop system. Agric Water Manag.

[R15] Chivenge P, Mabhaudhi T, Modi ATA, Mafongoya P (2015). The potential role of neglected and underutilised crop species as future crops under water scarce conditions in Sub-Saharan Africa. Int J Environ Res Public Health.

[R16] Choudhary S, Guha A, Kholova J, Pandravada A, Messina CD, Cooper M (2019). Maize, sorghum, and pearl millet have highly contrasting species strategies to adapt to water stress and climate change-like conditions. Plant Sci.

[R17] Confalonieri R, Orlando F, Paleari L, Stella T, Gilardelli C, Movedi E (2016). Uncertainty in crop model predictions: what is the role of users?. Environ Model Softw.

[R18] Dansi A, Vodouhè R, Azokpota P, Yedomonhan H, Assogba P, Adjatin A (2012). Diversity of the neglected and underutilized crop species of importance in benin. Sci World J.

[R19] Department of Agriculture Forestry and Fisheries (DAFF) (2003). Maize Production.

[R20] Dimes J, Revanuru S, Delve R, Probert M (2004). Evaluation of APSIM to simulate plant growth response to applications of organic and inorganic N and P on an Alfisol and Vertisol in India.

[R21] Duku C, Zwart SJ, Hein L (2018). Impacts of climate change on cropping patterns in a tropical, sub-humid watershed. PLoS ONE.

[R22] Eskandari H (2011). Intercropping of wheat (Triticum aestivum) and bean (Vicia faba): Effects of complementarity and competition of intercrop components in resource consumption on dry matter production and weed growth. African J Biotechnol.

[R23] Gashu D, Demment MW, Stoecker BJ (2019). Challenges and opportunities to the African agriculture and food systems. African J Food Agric Nutr Dev.

[R24] Gou F, van Ittersum MK, Simon E, Leffelaar PA, van der Putten PEL, Zhang L (2017). Intercropping wheat and maize increases total radiation interception and wheat RUE but lowers maize RUE. Eur J Agron.

[R25] Govender L, Pillay K, Siwela M, Modi A, Mabhaudhi T (2016). Food and nutrition insecurity in selected rural communities of KwaZulu-Natal, South Africa—linking human nutrition and agriculture. Int J Environ Res Public Health.

[R26] Granderson J, Price PN (2014). Development and application of a statistical methodology to evaluate the predictive accuracy of building energy baseline models. Energy.

[R27] Hadebe ST, Mabhaudhi T, Modi AT (2019). Water productivity of selected sorghum genotypes under rainfed conditions. Int J Plant Prod.

[R28] Hadebe ST, Modi AT, Mabhaudhi T (2017). Drought tolerance and water use of cereal crops: a focus on sorghum as a food security crop in Sub-Saharan Africa. J Agron Crop Sci.

[R29] Hammer GL, McLean G, Chapman S, Zheng B, Doherty A, Harrison MT (2014). Crop design for specific adaptation in variable dryland production environments. Crop Pasture Sci.

[R30] Hoffmann MP, Swanepoel CM, Nelson WCD, Beukes DJ, van der Laan M, Hargreaves JNG (2020). Simulating medium-term effects of cropping system diversification on soil fertility and crop productivity in southern Africa. Eur J Agron.

[R31] Holzworth D, Meinke H, DeVoil P, Wegener M, Huth N, Hammer G (2006). 3rd International Congress on Environmental Modelling And Software - Burlington, Vermont, USA - July 2006.

[R32] Holzworth DP, Huth NI, deVoil PG, Zurcher EJ, Herrmann NI, McLean G (2014). APSIM – Evolution towards a new generation of agricultural systems simulation. Environ Model Softw.

[R33] Hussain J, Khaliq T, Ahmad A, Akhtar J (2018). Performance of four crop model for simulations of wheat phenology, leaf growth, biomass and yield across planting dates. PLoS ONE.

[R34] Inthavong T, Tsubo M, Fukai S (2011). A water balance model for characterization of length of growing period and water stress development for rainfed lowland rice. F Crop Res.

[R35] Isaacs KB, Snapp SS, Chung K, Waldman KB (2016). Assessing the value of diverse cropping systems under a new agricultural policy environment in Rwanda. Food Secur.

[R36] Jamieson PD, Porter JR, Wilson DR (1991). A test of the computer simulation model ARCWHEAT1 on wheat crops grown in New Zealand. F Crop Res.

[R37] Jensen J, Bernhard R, Hansen S, McDonagh J, Møberg J, Nielsen N (2003). Productivity in maize based cropping systems under various soil-water-nutrient management strategies in a semi-arid, alfisol environment in East Africa. Agric Water Manag.

[R38] Jia J, Dai Z, Li F, Liu Y (2016). How will global environmental changes affect the growth of alien plants?. Front Plant Sci.

[R39] Jones JWW, Keating BA, Porter CHH (2001). Approaches to modular model development. Agric Syst.

[R40] Karunaratne AS, Azam-Ali SN, Al-Shareef I, Sesay A, Jørgensen ST, Crout NMJ (2010). Modelling the canopy development of bambara groundnut. Agric For Meteorol.

[R41] Keating BA, Carberry PS, Hammer GL, Probert ME, Robertson MJ, Holzworth D (2003). An overview of APSIM, a model designed for farming systems simulation. Eur J Agron.

[R42] Keatinge JDH, Wang JF, Dinssa FF, Ebert AW, Hughes JDA, Stoilova T (2015). Indigenous vegetables worldwide: their importance and future development. Acta Hortic.

[R43] Knörzer H, Lawes R, Robertson M, Graeff-Hönninger S, Claupein W (2011). Evaluation and performance of the APSIM crop growth model for German winter wheat, maize and fieldpea varieties in monocropping and intercropping systems. J Agric Sci Technol.

[R44] Kotir JH (2011). Climate change and variability in Sub-Saharan Africa: a review of current and future trends and impacts on agriculture and food security. Environ Dev Sustain.

[R45] Kunz RP, Davis NS, Thornton-Dibb S, Steyn JM, Jewitt G (2015). Assessment of Biofuel Feedstock Production in South Africa: Atlas of Water Use and Yield of Biofuel Crops in Suitable Growing Areas Report to the Water Research Commission.

[R46] Leff B, Ramankutty N, Foley JA (2004). Geographic distribution of major crops across the world. Global Biogeochem Cycles.

[R47] Mabhaudhi T, Chibarabada TP, Chimonyo V, Murugani V, Pereira L, Sobratee N (2019a). Mainstreaming underutilized indigenous and traditional crops into food systems: a South African perspective. Sustainability.

[R48] Mabhaudhi T, Chibarabada TP, Chimonyo VGP, Modi AT (2018a). Modelling climate change impact: a case of bambara groundnut (Vigna subterranea). Phys Chem Earth.

[R49] Mabhaudhi T, Chimonyo VGP, Hlahla S, Massawe F, Mayes S, Nhamo L (2019b). Prospects of orphan crops in climate change. Planta.

[R50] Mabhaudhi T, Chimonyo VGP, Modi AT (2017). Status of underutilised crops in South Africa: opportunities for developing research capacity. Sustainability.

[R51] Mabhaudhi T, Modi AT, Beletse YG (2013). Growth, phenological and yield responses of a bambara groundnut (Vigna subterranea *L. Verdc*) landrace to imposed water stress: II. Rain shelter conditions. Water SA.

[R52] Mabhaudhi T, Mpandeli S, Nhamo L, Chimonyo VGPP, Nhemachena C, Senzanje A (2018b). Prospects for improving irrigated agriculture in Southern Africa: linking water, energy and food. Water.

[R53] Malik AA, Chaudhary G, Khoobchandani M, Saxena A (2019). Biotechnology Products in Everyday Life.

[R54] Mangani R, Tesfamariam E, Bellocchi G, Hassen A (2018). Modelled impacts of extreme heat and drought on maize yield in South Africa. Crop Pasture Sci.

[R55] Martin-Guay M-O, Paquette A, Dupras J, Rivest D (2018). The new green revolution: sustainable intensification of agriculture by intercropping. Sci Total Environ.

[R56] Massawe F, Mayes S, Cheng A (2016). Crop diversity: an unexploited treasure trove for food security. Trends Plant Sci.

[R57] Matthews N, McCartney M (2018). Opportunities for building resilience and lessons for navigating risks: dams and the water energy food nexus. Environ Prog Sustain Energy.

[R58] Mayes S, Ho WK, Chai HH, Gao X, Kundy AC, Mateva KI (2019). Bambara groundnut: an exemplar underutilised legume for resilience under climate change. Planta.

[R59] McCown RL, Hammer GL, Hargreaves JNG, Holzworth DP, Freebairn DM (1996). APSIM: a novel software system for model development, model testing and simulation in agricultural systems research. Agric Syst.

[R60] McGregor JL (2005). C-CAM: Geometric Aspects and Dynamical Formulation.

[R61] McGregor JL, Dix MR (2001). The csiro conformal-cubic atmospheric GCM. Fluid Mech Appl.

[R62] McGregor JL, Dix MR (2008). High Resolution Numerical Modelling of the Atmosphere and Ocean.

[R63] Midega CAO, Bruce TJA, Pickett JA, Pittchar JO, Murage A, Khan ZR (2015). Climate-adapted companion cropping increases agricultural productivity in East Africa. F Crop Res.

[R64] Minda TT, van der Molen MK, Struik PC, Combe M, Jiménez PA, Khan MS (2018). The combined effect of elevation and meteorology on potato crop dynamics: a 10-year study in the Gamo Highlands, Ethiopia. Agric For Meteorol.

[R65] Missio JC, Rivera A, Figàs MR, Casanova C, Camí B, Soler S (2018). A comparison of landraces vs. modern varieties of lettuce in organic farming during the winter in the mediterranean area: an approach considering the viewpoints of breeders, consumers, and farmers. Front Plant Sci.

[R66] Mitchell SR, Emanuel RE, McGlynn BL (2015). Land–atmosphere carbon and water flux relationships to vapor pressure deficit, soil moisture, and stream flow. Agric For Meteorol.

[R67] Mpandeli S, Naidoo D, Mabhaudhi T, Nhemachena C, Nhamo L, Liphadzi S (2018). Climate change adaptation through the water-energy-food nexus in Southern Africa. Int J Environ Res Public Health.

[R68] Mrema GC, Kienzle J, Mpagalile J (2018). Current Status and Future Prospects of Agricultural Mechanization in Sub-Saharan Africa [SSA]. AMA, Agric Mech Asia, Africa Lat Am.

[R69] Muhammad YY, Mayes S, Massawe F (2016). Effects of short-Term water deficit stress on physiological characteristics of Bambara groundnut (Vigna subterranea (L.) Verdc. South African J Plant Soil.

[R70] Muzari W, Gatsi W, Muvhunzi S (2012). The impacts of technology adoption on smallholder agricultural productivity in Sub-Saharan Africa: a review. J Sustain Dev.

[R71] Nelson R, Dimes J, Silburn D, Paningbatan E, Cramb R (1998a). Erosion/productivity modelling of maize farming in the Philippine uplands - Part I. Agric Syst.

[R72] Nelson RA, Dimes JP, Paningbatan EP, Silburn DM (1998b). Erosion/productivity modelling of maize farming in the Philippine uplands - Part II. Agric Syst.

[R73] New Partnership for Africa’s Development (NEPAD) (2014). Implementation Strategy and Roadmap to Achieve the Vision on CAADP: Operationalizing the 2014 Malabo Declaration on Accelerated African Agricultural Growth and Transformation for Shared Prosperity and Improved Livelihood.

[R74] Nhamo L, Mathcaya G, Mabhaudhi T, Nhlengethwa S, Nhemachena C, Mpandeli S (2019). Cereal production trends under climate change: impacts and adaptation strategies in Southern Africa. Agriculture.

[R75] Nouri H, Stokvis B, Galindo A, Blatchford M, Hoekstra AY (2019). Water scarcity alleviation through water footprint reduction in agriculture: the effect of soil mulching and drip irrigation. Sci Total Environ.

[R76] O’Leary GJ, Aggarwal PK, Calderini DF, Connor DJ, Craufurd P, Eigenbrode SD (2018). Challenges and responses to ongoing and projected climate change for dryland cereal production systems throughout the world. Agronomy.

[R77] Padulosi S, Heywood V, Hunter D, Jarvis A, Yadav SS, Redden RJ, Hatfield JL, Lotze-Campen H, Hall AE (2011). Crop Adaptation to Climate Change.

[R78] Paff K, Asseng S (2018). A review of tef physiology for developing a tef crop model. Eur J Agron.

[R79] Peake AS, Robertson MJ, Bidstrup RJ (2008). Optimising maize plant population and irrigation strategies on the darling downs using the APSIM crop simulation model. Aust J Exp Agric.

[R80] Ran H, Kang S, Li F, Du T, Ding R, Li S (2017). Responses of water productivity to irrigation and N supply for hybrid maize seed production in an arid region of Northwest China. J Arid Land.

[R81] Rezvani Moghaddam P, Moradi R, Mansoori H (2014). Influence of planting date, intercropping and plant growth promoting rhizobacteria on cumin (Cuminum cyminum L.) with particular respect to disease infestation in Iran. J Appl Res Med Aromat Plants.

[R82] Rippke U, Ramirez-Villegas J, Jarvis A, Vermeulen SJ, Parker L, Mer F (2016). Timescales of transformational climate change adaptation in sub-Saharan African agriculture. Nat Clim Chang.

[R83] Saharan K, Schütz L, Kahmen A, Wiemken A, Boller T, Mathimaran N (2018). Finger millet growth and nutrient uptake is improved in intercropping with pigeon pea through “biofertilization” and “bioirrigation” mediated by arbuscular mycorrhizal fungi and plant growth promoting rhizobacteria. Front Environ Sci.

[R84] Saxena KB, Choudhary AK, Saxena RK, Varshney RK (2018). Breeding pigeonpea cultivars for intercropping: synthesis and strategies. Breed Sci.

[R85] Schlenker W, Roberts MJ (2009). Nonlinear temperature effects indicate severe damages to U.S crop yields under climate change. Proc Natl Acad Sci USA.

[R86] Schulze R (2011). Perspective on Climate Change and the South African Water Sector.

[R87] Schulze R, Horan M, Kunz R, Lumsden T, Knoesen D, Schulze R, Hewitson B, Barichievy K, Tadross M, Kunz R, Horan M (2011). Methods 2: Development of the Southern African Quinary Catchments Database. WRC Report No 1562/1/10.

[R88] Schulze RE, Chapman RD (2007). Estimation of Daily Solar Radiation Over South Africa. South African Atlas Climatol Agrohydrology WRC Rep.

[R89] Seyoum S, Chauhan Y, Rachaputi R, Fekybelu S, Prasanna B (2017). Characterising production environments for maize in eastern and southern Africa using the APSIM Model. Agric For Meteorol.

[R90] Slabbert R, Spreeth M, Krüger GHJ, Bornman CH (2004). Drought tolerance, traditional crops and biotechnology: breeding towards sustainable development. South African J Bot.

[R91] Smith A, Snapp S, Dimes J, Gwenambira C, Chikowo R (2016). Doubled-up legume rotations improve soil fertility and maintain productivity under variable conditions in maize-based cropping systems in Malawi. Agric Syst.

[R92] Snapp SSS, Grabowski P, Chikowo R, Smith A, Anders E, Sirrine D (2018). Maize yield and profitability tradeoffs with social, human and environmental performance: Is sustainable intensification feasible?. Agric Syst.

[R93] Soni ML, Yadava ND, Talwar HS, Nathawat NS, Rathore VS, Gupta K (2015). Variability in heat tolerance in Bambara groundnut (*Vigna subterranea* (L.) Verdc. Indian J Plant Physiol.

[R94] Sprent JI, Odee DW, Dakora FD (2010). African legumes: a vital but under-utilized resource. J Exp Bot.

[R95] Tcoli C (2016). Informal Food Systems and Food Security in Rural and Urban East Africa.

[R96] Tokatlidis I, Vlachostergios D (2016). Sustainable stewardship of the landrace diversity. Diversity.

[R97] Ukeje E (2010). Modernizing Small Holder Agriculture to Ensure Food Security and Gender Empowerment: Issues and Policy.

[R98] Vadez V, Berger JD, Warkentin T, Asseng S, Ratnakumar P, Rao KPC (2012). Adaptation of grain legumes to climate change: a review. Agron Sustain Dev.

[R99] van Ittersum MK, van Bussel LGJ, Wolf J, Grassini P, van Wart J, Guilpart N (2016). Can sub-Saharan Africa feed itself?. Proc Natl Acad Sci USA.

[R100] Xiao D, Liu DL, Wang B, Feng P, Bai H, Tang J (2020). Climate change impact on yields and water use of wheat and maize in the North China Plain under future climate change scenarios. Agric Water Manag.

[R101] Xie H, Huang Y, Chen Q, Zhang Y, Wu Q (2019). Prospects for agricultural sustainable intensification: a review of research. Land.

[R102] Yu Y, Stomph TJ, Makowski D, Zhang L, van der Werf W (2016). A meta-analysis of relative crop yields in cereal/legume mixtures suggests options for management. F Crop Res.

[R103] Zhao C, Liu B, Piao S, Wang X, Lobell DB, Huang Y (2017). Temperature increase reduces global yields of major crops in four independent estimates. Proc Natl Acad Sci USA.

