# Supplementary material for: Optimizing Traditional Cropping Systems Under Climate Change: A Case of Maize Landraces and Bambara Groundnut
Source: Front Sustain Food Syst. Author manuscript; Available in PMC 2024 Jul 19. (PMC7616261; doi:10.3389/fsufs.2020.562568)
Supplement: Supplementary material [file EMS197509-supplement-Supplementary_material.pdf]

Supplementary information 1: Water use and water use efficiency of maize landrace – bambara groundnut – dry bean intercrop system

1.0 Introduction

Maize (*Zea mays* L.) is the staple food crop in South Africa (van Averbeke et al., 2011) and is grown by many smallholder farmers. However, many smallholder farming systems are typified by low maize yields as a result of socio-economic and biophysical constraints (Ortmann and King, 2010). Among these, water availability has been noted to be the most critical yield-limiting factor (TerAvest et al., 2015). The 2014/15 and 2015/16 drought, which was the worst since the start of record-keeping in 1901 caused widespread yield losses and crop failure (Meyer et al., 2016) for rainfed maize systems. Smallholder farmers, the majority of whom reside in already marginal areas, were particularly affected. While water stress is the primary limiting factor, it has also been observed that lack of access to improved maize varieties that are best suited to farmers' environments contributes to low yields (Van Averbeke et al., 2011; Van Averbeke and Khosa, 2007; Wenhold et al., 2007). In this regard, maize landraces have been identified as a genetic resource with potential to improve low-input low-output smallholder farmer systems in marginal environments.

Maize landraces can be defined as domesticated, locally adapted and traditional maize varieties that have been developed over hundreds of years, through adaptation to the natural and cultural environment due to natural and farmer selection (Zeven, 1998). Therefore, maize landraces have undergone significant natural and artificial selection making them highly adaptable to harsh environments under which smallholder farmers reside (Aguiriano et al., 2008). Several reports have shown that maize landraces possess heat (Driedonks et al., 2016; Ncube et al., 2011) and drought (Bazargani et al., 2011) tolerance. Although low yielding relative to improved genotypes, yields are generally stable under low resource availability (Hellin et al., 2014). For instance, Oliveira et al. (2013) observed that maize landraces of different provenance were adaptable and stable across different environments in Mexico. Such

attributes make maize landraces ideal for sustainable production in low-input low-output systems. However, the fact that they yield less than hybrids under optimal conditions acts as a disincentive for their adoption in interventions to improve agricultural productivity and food security, and drive rural development (Modi and Mabhaudhi, 2013). It has been suggested that lack of knowledge on their agronomy and best management practices could be contributing to their current status as underutilized crops (Mabhaudhi et al., 2017). Poor agronomic and water management strategies combined with water stress in poor rainfall areas has been associated with observed low yields (Botha et al., 2015). In this regard, several opportunities exist for increasing water productivity of maize landraces under limited water availability. One such strategy is intercropping maize landraces with legumes.

Intercropping is defined as a traditional form of agriculture were two or more crops are grown on the same piece of land varying in either spatial and/or temporal resolution (Willey, 1979). Under complimentary interactions, intercropping has been observed to increase crop yields per unit area, and overall system yield, with a fixed amount of water entering the system relative to monocropping. This has been attributed to (i) an increase in the efficiency of capture and use of available soil water (Chimonyo, 2016; Mabhaudhi and Modi, 2014), (ii) reduction in unproductive loss of water from bare soil evaporation and runoff (Gao et al., 2013), (iii) increased agro-biodiversity which improves yield stability under varying climatic conditions (Thrupp, 2000), and (iv) increase in overall yield per unit area relative to monocrop systems (Naim et al., 2013). Intercropping maize landraces with legumes could result in improved resource capture and utilization and hence improved productivity. Cereal-legume intercrop systems present a sustainable technology that can improve food crop diversity and system stability; thus improving short to long term food and nutrition security (Chikowo et al., 2014). In addition, such intercrop systems can aid in improving soil integrity after several cycles; making the technique ideal for the rehabilitation of degraded soils that characterize smallholder farming lands (Jun et al., 2014; Nduku, 2014; Sujatha and Bhat, 2010; Wang et al., 2014; Wise et al., 2007). Intercropping presents a sustainable coping strategy that could lead to long term adaptation to climate change and variability (Chimonyo et al., 2015).

Despite these positive prospects, the promotion of intercropping, especially with maize landraces systems has been limited within low-input low-output smallholder farming systems in marginal lands. This could be due to limited information quantifying productivity of maize landrace intercrop systems, their water use and subsequent WUE. To ensure the successful and sustainable promotion of maize landrace and their legume intercrop systems, there is a need to quantify productivity and resource use efficiencies. In this study, it was hypothesized that intercropping maize with either dry bean (*Phaseolus vulgaris*) or bambara groundnut (*Vigna subterranea* (L.) Verdc.) could improve agricultural output and increase water use efficiency. The objectives of the study were to (i) quantify the productivity of maize – dry bean – bambara groundnut intercrop systems under different water regimes, and (ii) quantify water use and determine water use efficiency of maize – dry bean – bambara groundnut intercrop systems.

2.0 Materials and methods

2.1 Plant material

Three species were used in this study, namely, maize landraces (*Zea mays* L.), dry bean (*Phaseolus vulgaris)* and bambara groundnut (*Vigna subterranea* (L.) Verdc.). The maize landrace was sourced from local farmers in Gqunge, Eastern Cape Province, South Africa. The landrace is an early to medium (90 – 120 days) maturing variety with a yield potential of 3 t ha^{-1} (Mazvimbakupa, 2014). Its plant height has been observed to range between 150 – 180 cm, making it ideal for intercropping. A bambara groundnut landrace was sourced from Pongola, KwaZulu-Natal, South Africa (27.3831° S, 31.6198° E). The landrace is a medium to late (120 – 150 days) maturing variety with a yield potential of 0.5 – 2 t ha^{-1} (Mabhaudhi et al., 2013). Information regarding its use in intercrops is limited. A dry bean variety, Ukulinga, was sourced from McDonald Seeds, Pietermaritzburg, South Africa. Ukulinga is a determinate early to medium (90 – 120 days) maturing variety with a yield potential of about 2 – 5 t ha^{-1}.

2.2 Site description

A field trial was conducted at the University of KwaZulu–Natal's Ukulinga Research Farm (29°40'S; 30°24'E; 809 m a.s.l.) during the 2015/16 planting season. Ukulinga Research Farm is classified as semi-arid with 77% of the mean annual rainfall of 750 mm received mostly between the months of October and April. The summer months are warm to hot with an average temperature of 26.5°C while temperatures as low as 8.0°C have been observed during winter (Kunz et al., 2015).

The soils are characterized as predominantly clay to clay–loam soils and are moderately shallow ranging from 0. 6 m to 1 m. Based on soil texture, the soil water characteristics (bulk density (g m^{-3}), hygroscopic water content (mm m^{-1}), permanent wilting point (mm m^{-1}), field capacity (mm m^{-1}) total available water (mm m^{-1}), saturation (mm m^{-1}) hydraulic conductivity (mm hr^{-1}) were all determined using hydraulic properties calculator (http://hydrolab.arsusda.gov/soilwater/Index.htm) (Supplementary Table 1). Results of soil chemical properties showed that the carbon (%) for the top 0.2 m layer was 3.5% while N was 0.35%. From these the initial C:N ratio was calculated as 10.

Supplementary Table 1: Soil water properties at different depths for soil at the experimental site.

Texture	BD[1]	HC[2]	PWP[3]	FC[4]	TAW[5]	SAT[6]	K_{SAT}[7]
	g m^{-3}	--------------------------mm m^{-1}------------------------					mm h^{-1}
Clay	1.35	0.33	294	416	152	489	19,70

[1] Bulk density; [2] Hygroscopic moisture content; [3] Permanent wilting point; [4] Field capacity; [5] Total available water; [6] Saturation; [7] Hydraulic conductivity.

2.3 Experimental design and layout

The experimental design was a split–plot design with sub-plots laid out in randomized complete blocks within the main plots and replicated three times. The main plot was water regime with two levels (irrigation and rainfed). Sub–plots comprised intercrop combinations, with five intercrop combinations. To ensure good establishment across all the treatments, the trial was established under irrigation. Irrigation was withdrawn

at establishment for treatments grown under rainfed conditions. Crop establishment was defined as when 90% of experimental plants had emerged.

Water regimes: There were two water regimes – irrigated and rainfed. Full irrigation involved watering crops up to 100% of maize water requirement for the duration of the trial. Irrigation scheduling was based on crop water requirement calculated from the product of maize crop factors (K_c) (Allen et al., 1998) and Priestley-Taylor (PT) reference evapotranspiration (ET_o) values obtained from an automatic weather station (AWS) located within a 1 km radius from the experimental field. The K_c values for grain maize were K_c initial = 0.30 (25 days), K_c mid = 1.20 (70 days), and K_c end = 0.35 (45 days). The durations in brackets indicate the corresponding periods in days (total of 140) for which the crop factors were applied. Crop water requirement (ET_c) was calculated as described by Allen et al. (1998):

$$ET_c = ET_o * K_c \qquad \text{Equation 1}$$

where: ET_c = crop water requirement in mm, ET_o = reference evapotranspiration in mm, and K_c = crop factor.

Irrigation scheduling was done weekly (every 7 days) and applied using a sprinkler system. Within the seven-day period and in the event of rainfall, irrigation scheduling was adjusted accordingly. The amount of water applied at each irrigation event amount was recorded using rain gauges randomly placed within the experimental plots. During the growing period, supplementary irrigation applied in the FI treatment was 76 mm and cumulative rainfall was 288.81.

Intercrop treatment: The component crops were maize landraces, bambara groundnut and dry bean. The intercropping treatments were: maize landrace (sole), bambara groundnut (sole), dry bean (sole), maize landrace + bambara groundnut (intercrop) and maize landrace + dry bean (intercrop).

Intercropping systems were designed as additive intercrop systems. Since dominant cropping systems in semi-arid areas are maize-mixed (Cairns et al., 2013), the maize landrace was considered as the main crop and was sown at 100% of its recommended plant population in pure and intercrop stands. Bambara groundnut and dry bean were then "added" to the maize landrace by planting additional rows between rows of maize.

Individual plot sizes for each treatment were an area of 13.5 m^2. All rows were 4.5 m long and inter-row spacing for maize landrace (sole and intercrop treatment) and sole bambara groundnut and sole dry bean was 0.75 m. For the intercrop treatments, rows for intercrops were made in the middle (0.375 m) of maize rows. An in-row spacing of 0.50 m was used for maize. For sole bambara groundnut and dry bean, in-row spacing was 0.30 m. Under intercropping, the in-row spacing was maintained at 0.30 m. Plant populations of the maize landrace, bambara groundnut and dry been were 26 666, 44 444 and 44 444 plants ha^{-1} for both sole and intercrop treatments. The plant population used for maize landrace, the main crop component were based on recommended densities for dryland maize production (Jensen et al., 2003).

2.4 Data collection

Climate data: Daily weather data were obtained from an automatic weather station (AWS) located less than 1 km from the experimental field and within Ukulinga Research Farm. The AWS is part of the Agricultural Research Council – Institute for Soil, Climate and Water (ARC–ISCW) network of automatic weather stations. Daily weather parameters that were collected included: maximum (T_{max}) and minimum (T_{min}) air temperature (°C), solar radiation (Rad, MJ m^{-2}), rainfall (mm) and PT- ET_o (mm).

Crop growth, physiology and yield: Crop data collected included phenological stages such as times to emergence, end of juvenile stage, end of vegetative stage, floral initiation, flowering, cob/pod formation, grain filling, physiological maturity and harvest maturity. A phenological event was deemed to have occurred when it was observed in at least 50% of experimental plants. Observations of crop phenology were recorded in calendar days and later converted to thermal time using method 2 as described by McMaster and Wilhelm (1997). Measurements of plant height (PHT), leaf number (LN), leaf area index (LAI), stomatal conductance (g_s), chlorophyll content index (CCI), leaf chlorophyll fluorescence (Fv/Fm) and biomass accumulation were collected on a weekly basis for all component crops. At physiological maturity of the maize landrace, all component crops were harvested. Yield and yield components (cob/pod number per plant, grain number per pod, grain weight per cob/pod, 1000 grain weight, harvest index (HI) and yield) were then determined. The trial was

harvested at physiological maturity since maize cobs and bambara groundnut pods were being attacked by monkeys.

Productivity of the intercrop systems was evaluated using Land Equivalent Ratio (LER) as described by Willey (1979).

$$LER = L_a + L_b = \frac{Y_a}{S_a} + \frac{Y_b}{S_b} \qquad \text{Equation 2}$$

where: LER = land equivalent ratio, L_A and L_b = LERs of component crop *a* (maize), and *b* (dry bean or bambara groundnut), respectively, and Y_a and Y_b represent intercrop yield component crop *a* (maize), and *b* (dry bean or bambara groundnut), respectively, while S_a and S_b are their respective sole.

Water use: Water use (ET) for each treatment was calculated as the residual of a soil water balance:

$$ET = P + I - D - R - \Delta SWC \qquad \text{Equation 3}$$

where: ET = evapotranspiration (mm), P = precipitation/rainfall (mm), I = irrigation (mm), D = drainage (mm), R = runoff (mm), and ΔSWC = changes in soil water content (mm). Runoff (R) was assumed to be zero since it was negligible in the plots as they had a slope of less than 5%. Drainage was also considered negligible since the observed impeding layer at 0.6 m restricted downward movement of water beyond the root zone. Another reason for rendering drainage negligible was that the top 0.6 m depth was never observed to be reaching field capacity.

Changes in soil water content (SWC) were measured using a PR2/6 profile probe connected to an HH2 handheld moisture meter (Delta–T, UK). The soil profile at Ukulinga is shallow with an effective rooting depth of 0.60 m (Supplementary Table 1). The PR2/6 profile probe has sensors positioned at 0.10, 0.20, 0.30, 0.40, 0.60 and 1.00 m along the probe. Sensors used in the analysis of SWC where the first 5 (0.10 – 0.60). Due to small variations occurring at depths of 0.20 and 0.30 m and 0.40 and 0.60 m, respectively, results for SWC were only presented for depths of 0.10, 0.30 and 0.60 m. Weekly rainfall (R) was obtained from data obtained from the AWS.

To determine whether intercropping resulted in changes in water use, the following equation suggested by Morris and Garrity (1993) was used:

$$\Delta WU\ (\%) = \left[\left(\frac{WU_{ic}}{P_a WU_{sa} + P_b WU_{sb}}\right) - 1\right] * 100\% \qquad \text{Equation 4}$$

where: WU_{ic}, WU_{sa} and WU_{sb} = the water use in intercropping, sole cropping species A and sole cropping species B, respectively, and Pa and Pb are the proportions of species A and B in the intercrop, given by Pa = Da/(Da + Db) with Da and Db being the density in intercropping relative to sole cropping of species A and B, respectively.

Water use efficiency: Water use efficiency was only calculated for the sole treatments since it was not possible to separate water use for each component crop in the intercrop systems. Water use efficiency of sole cropping system was therefore calculated as follows:

$$WUE_{Y/B} = \frac{Y/B}{WU}\,(kg\ mm^{-1}\ ha^{-1}) \qquad \text{Equation 5}$$

where: WUE = water use efficiency (kg mm^{-1} ha^{-1}) and Y = the economic yield (kg ha^{-1}), B = final biomass (kg $ha^{-1)}$ and ET = the water use (mm).

To determine whether intercropping resulted in changes in water use efficiency the following equation suggested by Morris and Garrity (1993) was used:

$$\Delta WUE\ (\%) = \left(\frac{\frac{Y_{ic}}{WU_{ic}}}{\left(\frac{P_a Y_{sa}}{WU_{sa}}\right) + \left(\frac{P_b Y_{sb}}{WU_{sb}}\right)} - 1\right) * 100\% \qquad \text{Equation 6}$$

where: Yic, Ysa and Ysb = the yields in intercropping and sole cropping of species A and B, respectively.

For interpretation, when ∆WU and ∆WUE are greater than zero, WU and WUE are assumed to be higher in the intercrop system relative to the sole crop.

2.5 Agronomic management

Prior to planting, soil samples were obtained from the field trial site and analysed for soil fertility and textural analyses. Based on results of soil fertility analyses, a compound fertilizer with a N:P:K ratio of 2:3:2 (22) was applied to supply 15 kg N ha^{-1}. Fertilizer application was designed to meet the nutritional requirements for maize, the main crop, and was broadcast at planting.

Land preparation involved ploughing, disking and rotovating to achieve fine tilth. Planting was done by hand; planting depth for all crops ranged from 2–3 cm. For maize, rows were opened and seed sown within the rows. Upon full establishment (90% emergence), the maize landrace was thinned to the required spacing; excess seedlings were used for gap–filling. Routine weeding was done using hand hoes. Insect pests and animal attacks were scouted for at each visit to the field

2.6 Data analysis

Data collected was subjected to analysis of variance (ANOVA) using GenStat® (Version 16, VSN International, UK) and means of significantly different variables separated using Fisher's unprotected.in GenStat® at the 5% level of significance.

3.0 Results

3.1 Weather and soil conditions

3.1.1 Weather

Weather data for the growing period was consistent with long term weather data for Ukulinga (Section 2.2). Overall, the average maximum temperature was 28.56 ± 5.00°C while the minimum temperature was 16.47 ± 2.24°C. Maximum temperature was 2°C higher than long term temperature averages of 26.5°C. A total of 29 days had above optimum temperatures (30°C) for the maize landrace growth suggesting higher GDD (°Cd), and this would suggest faster crop development. A total of 12 days, of which five occurred during tasselling stage, were considered to be extremely hot (> 35°C) days implying temperature stress could have occurred.

During the growing period, cumulative rainfall was 288.81 mm and the distribution was positively skewed to the early and mid-growing period. There were 75 days when no rain was recorded out of 107 days (Fig 1). During the growing period there were 8 dry spells of which 5 occurred during the last half. A dry spell was defined as a period of 5 consecutive days with rainfall of less than 7.5 mm. These results suggest that the possibility of intermittent water stress was observed towards the end of the growing period. Cumulative reference evapotranspiration was 348.27 mm which indicated a deficit of 59.46 mm from observed rainfall received.

The incidences of storm events were experienced twice during the growing period (24th January and 16th March, 2015) and coincided with the early and mid - vegetative growth stage for all three crops hence exposing plants to waterlogging, especially bambara groundnut (Supplementary Fig. 1). A storm event was defined as a rainfall event with an intensity of greater than 25 mm hr^{-1}.

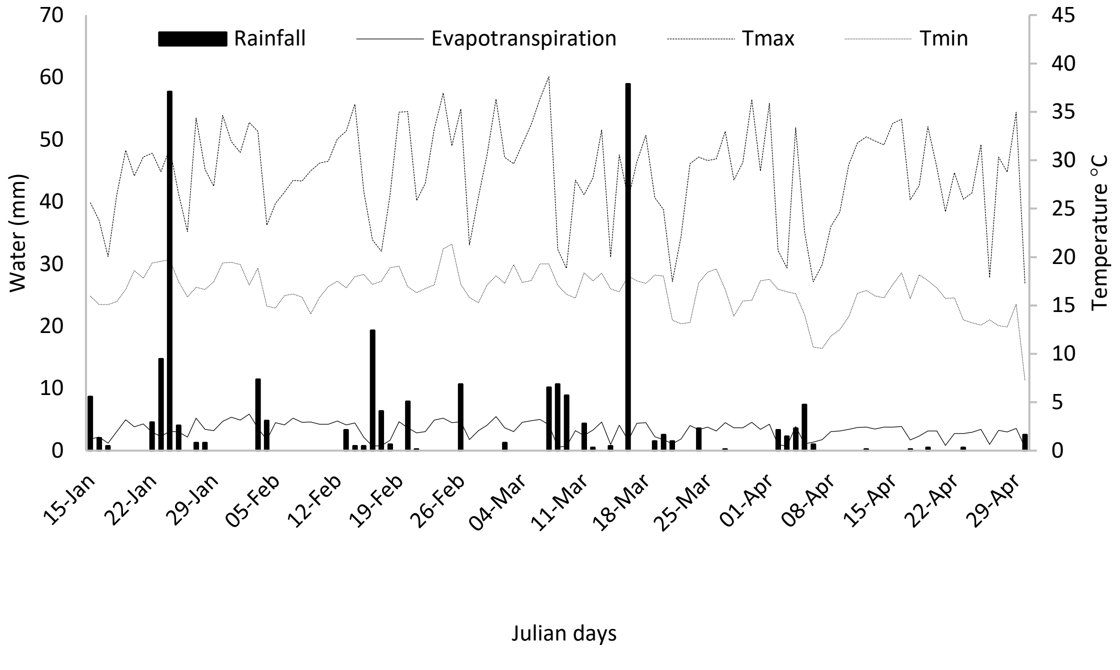

Supplementary Figure 1: Climate data (rainfall, reference evapotranspiration and minimum (Tmin) and maximum (Tmax) temperature) at Ukulinga during the growing period.

3.1.2 Soil water

Overall, soil water content (SWC) under irrigated conditions was 10.5% higher and more constant (±5.3) throughout the growing period when compared to rainfed conditions (Supplementary Fig 2). On average, total available water (TAW) under irrigated plots was 41mm ± 71 compared to 18 mm ± 52 under rainfed conditions. Under irrigation, plots of dry bean had the highest TAW (128 mm ± 36) relative to plots of bambara groundnut (85 mm ± 56). When comparing the maize landrace cropping systems under irrigated conditions, it was observed that TAW was 45% higher under intercropping relative to sole maize landrace (Supplementary Fig 2). A similar trend was observed under rainfed conditions; plots with intercropped maize landrace were observed to have higher TAW [maize – bambara groundnut (102 mm ± 42) and maize – dry bean (56 mm ± 56)] relative to those of sole maize landrace with SWC which was observed to by close to or below PWP. Intercropping maize landrace with either dry bean or bambara groundnut improved TAW by 56 and 100%, respectively, relative to sole cropped maize landrace (Supplementary Fig 2).

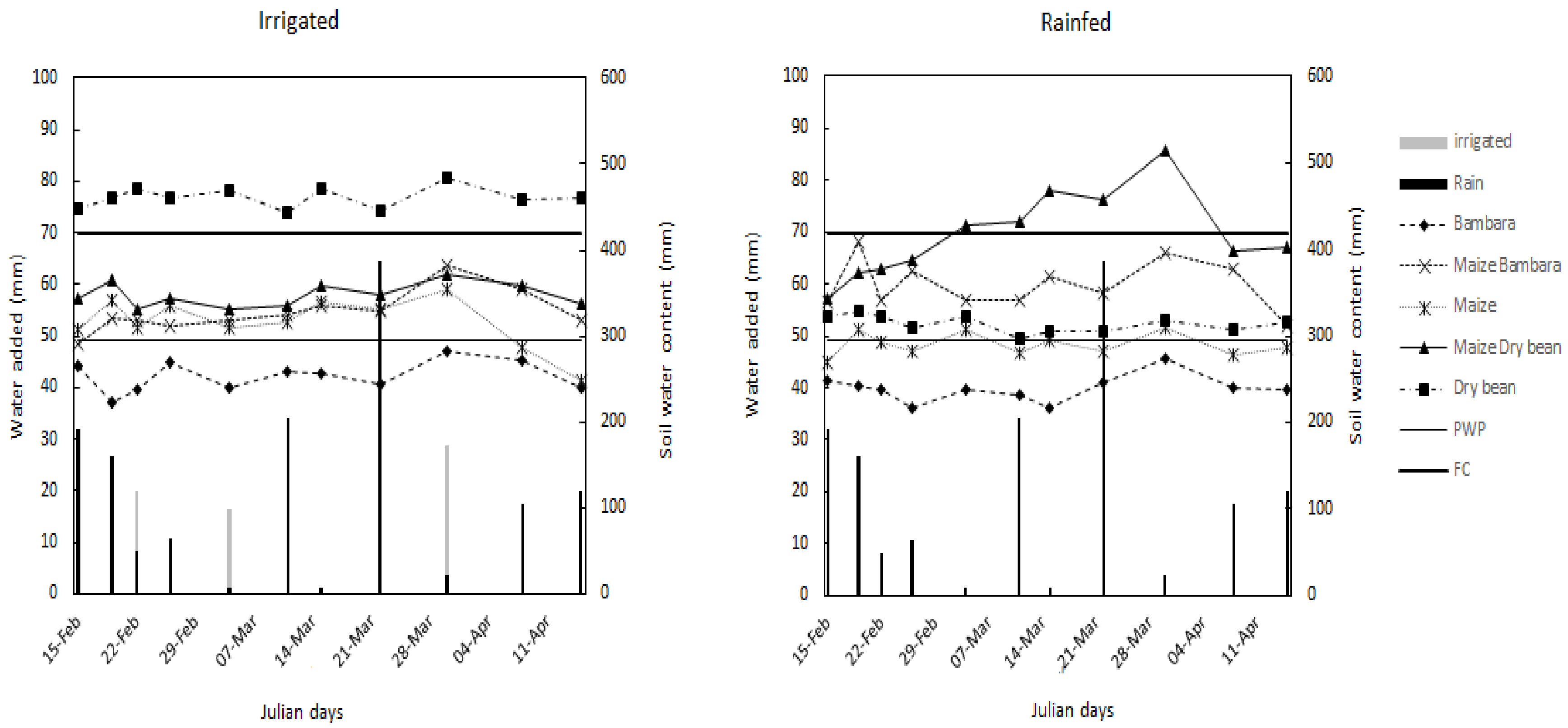

Supplementary Figure 2 Comparison of soil water content within a depth of 1 m in response to cropping systems (Maize, Bambara groundnut, Dry bean, Maize – Bambara groundnut, Maize – Dry bean) and water regimes (Irrigated and Rainfed).

3.2 Plant physiology and growth

For maize landrace grown under rainfed conditions, CCI and Fm/Fv were significantly ($P < 0.05$) lower when relative to irrigated conditions (Supplementary Fig. 3). This was consistent with the observed trend for TAW. Regardless of water regime, intercropping the maize landrace with dry bean resulted in low CCI and Fm/Fv relative to maize landrace intercropped with bambara groundnuts and sole cropped maize (Supplementary Fig. 3). These results are contrary to what was observed for LAI and TAW which were higher for maize landrace intercropped with dry bean relative to the other cropping systems.

Significant ($P < 0.05$) differences were observed for LAI across the maize landrace cropping systems over time. It was observed that, across the water regimes, intercropping maize landrace with either bambara groundnut or dry bean resulted in a significantly higher (31 and 62%, respectively) LAI relative to sole cropped maize landrace (Supplementary Fig. 4). This was attributed to the additive nature of the intercrop system where either bambara groundnut or dry bean were added into the maize landrace stand. While there were overall improvements of LAI for maize landrace intercropped with either bambara groundnut or dry bean, intercropping maize landrace with dry bean resulted in a LAI 50% higher than when the maize landrace was intercropped with bambara groundnuts. Results of LAI are consistent with the trend observed for TAW.

Significant ($P < 0.05$) differences were observed for maize landrace g_s under the different cropping systems over time (Supplementary Fig. 3). It was observed that, under irrigated conditions maize landrace g_s was high and stable across cropping systems The observed trend was sole maize (236 mmol m^{-2} s^{-1} ± 56) < maize – bambara groundnut (248 mmol m^{-2} s^{-1} ± 64) < maize – dry bean (252 mmol m^{-2} s^{-1} ± 36). This could be attributed to the higher TAW and increased frequency of wetting interval. Under rainfed conditions, intercropping maize landrace with either bambara groundnut (226 mmol m^{-2} s^{-1}) or dry beans (213 mmol m^{-2} s^{-1}) resulted in significantly higher g_s relative to sole cropped maize (189 mmol m^{-2} s^{-1}). Overall, the observed results of g_s across the maize landrace cropping systems and water regimes were consistent with the observed trends for TAW and LAI.

There were no significant differences for maize landrace plant growth parameters [leaf number, plant height and destructive leaf area index (LAI)] in response to cropping system or water regime. It could be that the growth parameters were not as sensitive to water and cropping systems as were physiological parameters.

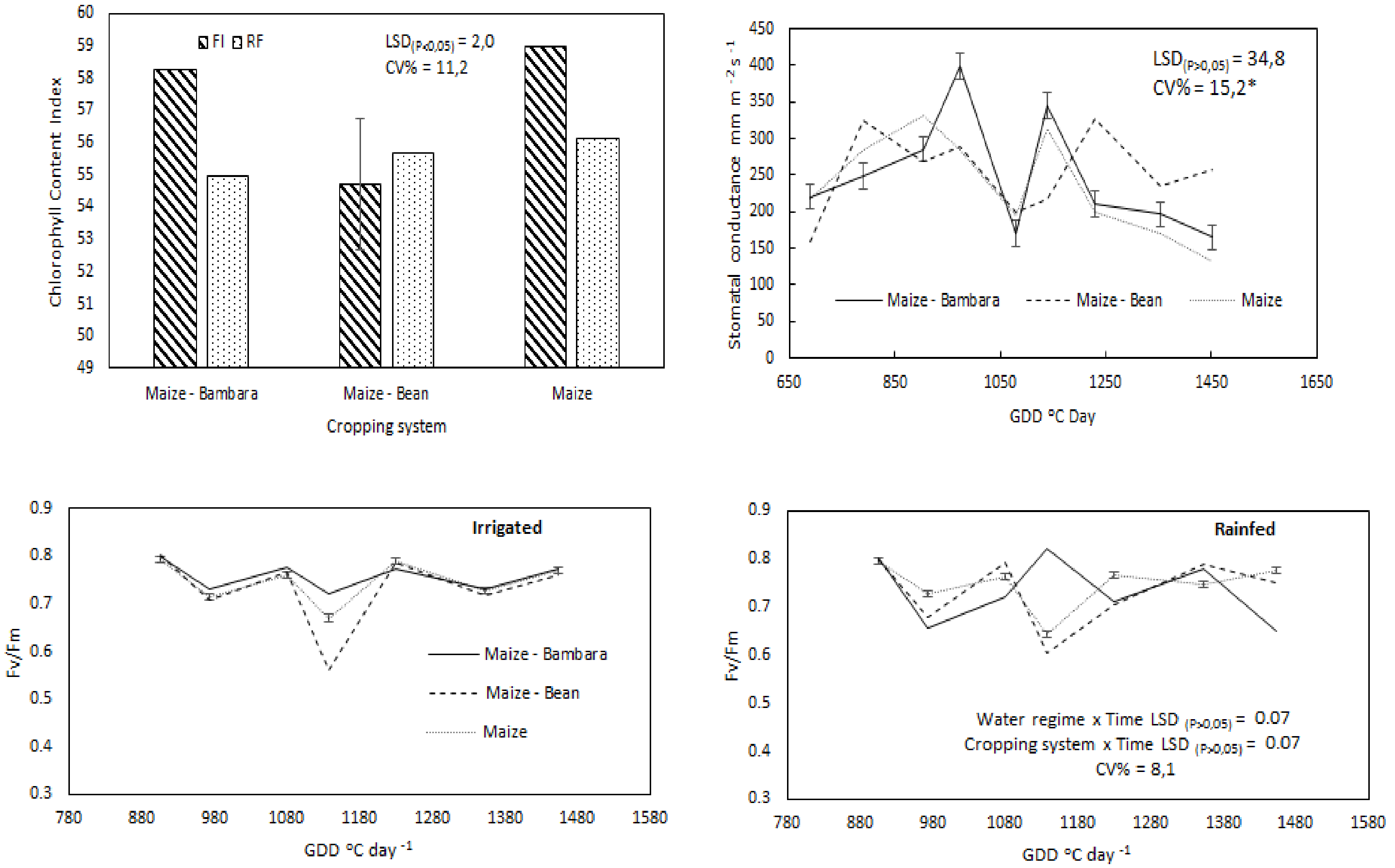

Supplementary Figure 3: Comparison of (i) chlorophyll content index and (ii) stomatal conductance (iii) leaf fluorescence in response to cropping systems (Maize, Bambara groundnut, Dry bean, Maize – Bambara groundnut, Maize – Dry bean) and water regimes (Irrigated and Rainfed).

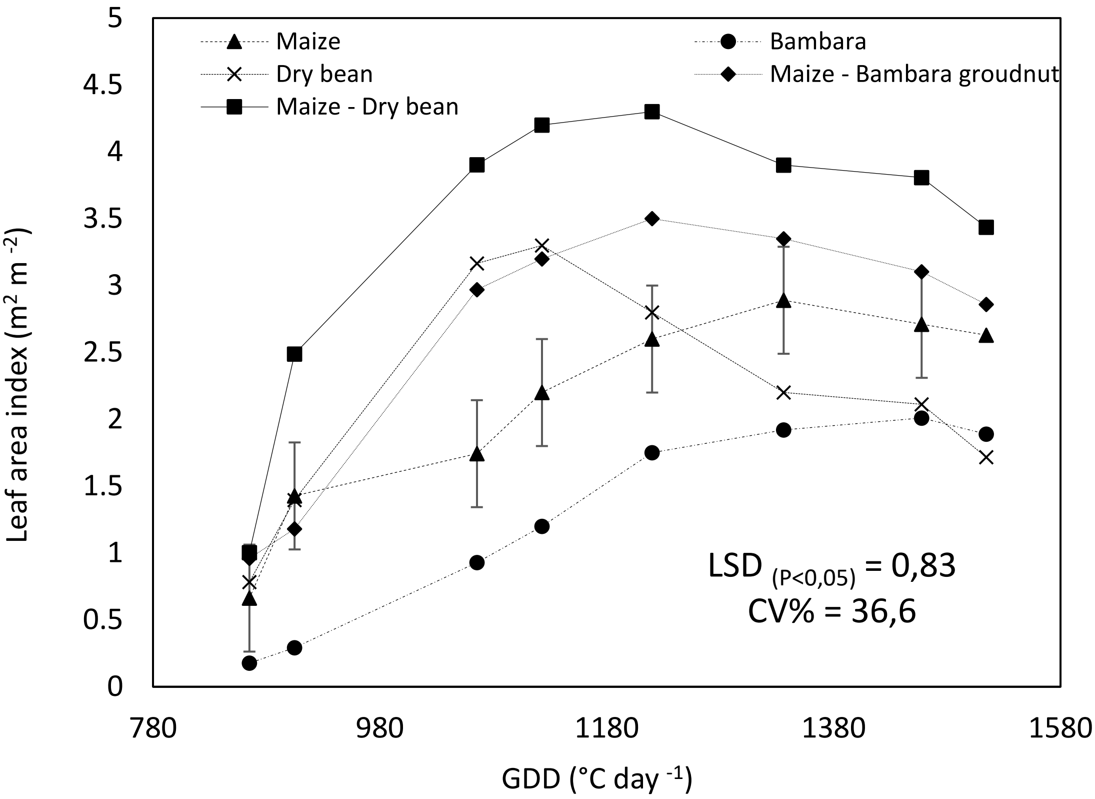

Supplementary Figure 4: Comparison of leaf area index (LAI) for different cropping systems (Sole- maize, bambara groundnut, dry bean, maize - bambara groundnut and maize – dry bean) over time

3.3 Yield and yield components

Water regime and intercropping did not have a significant effect on maize landrace yield (Supplementary Table 2) and this could be related to the insignificant effects on crop growth parameters (leaf number, LAI and PHT). On the other hand, significant (P< 0.05) differences were observed for yield and yield component responses to intercrop for the legume species. Despite the improvements in TAW under intercropping, yield of bambara groundnut and dry bean was lower by 41% and 56%, respectively, under intercropping relative to the sole crops.

The productivity of the maize landrace intercrop system was evaluated using land equivalent ratio. Although not statistically significant, results of LER showed that intercropping maize landraces with either bambara groundnut or dry bean resulted in 30% higher overall productivity across water regimes.

Supplementary Table 2: A comparison of biomass yields and harvest index for maize, bambara groundnut and dry bean in response to different cropping (Maize, Bambara groundnut, Dry bean, Maize – Bambara groundnut, Maize – Dry bean) and water regimes (Irrigated and Rainfed).

Water regime	Cropping system	Maize (M)			Bambara groundnut (B)			Dry bean (D)		
		Biomass	Yield	HI	Biomass	Yield	HI	Biomass	Yield	HI
Full irrigation	Sole Systems	2.48	0.83	0.30	1.77	0.45c[1]	0.25	2.86b	1.12bc	0.39
	M + B	2.47	0.87	0.40	0.95	0.15a	0.15	-	-	-
	M + D	2.56	0.72	0.30	-	-	-	1.32a	0.57ab	0.42
Rainfed	Sole Systems	2.51	0.73	0.30	1.67	0.47c	0.29	2.92b	1.27c	0.43
	M + B	2.37	0.82	0.30	1.06	0.23b	0.23	-	-	-
	M + D	2.57	0.79	0.30	-	-	-	1.04a	0.46a	0.45
Mean		2.46	0.80	0.3	1.39	0.32	0.25	2.03	0.86	-
$P_{(value)}$[2]		NS	NS	NS	NS	*	NS	**	*	NS
$LSD_{(P<0.05)}$		-	-	-	-	0.07	-	0.83	0.64	
CV%		-	-	-	-	19	-	24	35	-

[1] Means followed by the same letter indicate that they were not significantly different (p < 0.05) from each other; [2] * and ** significant difference at P < 0.01 and P < 0.05

3.4 Water use and water use efficiency

Overall, cropping systems grown under irrigation had higher water use (286 mm) relative to those grown rainfed (210 mm) conditions. This was consistent with the overall trend for TAW (Section 3.1.2 and 3.2). Under irrigated conditions, differences in WU between the intercropped maize landrace systems and sole cropped maize landrace were nominal (4 mm). Intercropping maize landraces with dry bean improved WU by 7.5% relative to sole cropped maize landraces and dry beans, respectively. A decrease in WU (-1.5%) was observed when the maize landrace was intercropped with bambara groundnut. Observed WU for maize landrace intercropped with dry bean was consistent with trends for TAW, g_s and LAI. Observed WU for maize landrace intercropped with bambara groundnut was inconsistent with trends for TAW and g_s.

Under rainfed conditions, intercropping maize landrace with either bambara groundnut or dry bean resulted in lower WU (31 and 11%, respectively) relative to sole cropped maize landrace. This was inconsistent with observed higher TAW observed for intercropped maize landrace relative to sole cropped maize landrace under rainfed conditions. Intercropping maize landrace with dry bean resulted in the highest improvements in WU of 26% relative to sole cropped maize landrace and dry beans, respectively. This was consistent with observed trends for LAI. Intercropping maize with bambara groundnut resulted in a reduction in WU (-24%) relative to sole cropped component crops. This was similar to what was observed under irrigated conditions.

Overall, water use efficiency was higher (41%) under rainfed conditions than under irrigated conditions. Dry bean had the highest WUE (5.7 kg mm^{-1} ha $^{-1}$) across all water regimes and this was followed by maize landrace (3.2 kg mm^{-1} ha $^{-1}$) and bambara groundnut (1.9 kg mm^{-1} ha $^{-1}$). High WUE observed for dry bean was consistent with results of low WU and higher yields obtained. Intercropping maize landrace with bambara groundnut improved WUE (77%) regardless of water regime. This was attributed to the low WU in the maize – bambara groundnut intercrop systems relative to sole cropped components. On the other hand, intercropping the maize landrace with dry bean resulted in the least improvements of WUE regardless of water regime. This was attributed to the high WU in the maize – dry bean intercrop systems relative to sole cropped components.

Supplementary Table 3: A comparison of water use and water use efficiency across different cropping systems [maize (sole), bambara groundnut (sole), dry bean (sole), maize - bambara groundnut (intercrop) and maize - dry bean (intercrop]) and response to different water regimes

Water regime	Cropping system	Yield of sole crop (t ha^{-1})	Yield of intercrop (t ha^{-1})	System water use (mm)	Improvements in WU (%)	WUE (kg mm^{-1} ha^{-1})	Improvements of WUE (%)
Irrigation	Bambara groundnut	0.5	–	297	–	1.5	–
	Dry bean	1.1	–	251	–	4.5	–
	Maize	0.9	–	293	–	3.0	–
	Maize – Bambara groundnut	0.9	0.2	291	-1.2	–	66.6
	Maize - Dry bean	0.9	0.5	297	7.5	–	15.8
Rainfed	Bambara groundnut	0.5	–	199	–	2.6	–
	Dry bean	1.3	–	185	–	6.8	–
	Maize	0.9	–	259	–	3.5	–
	Maize – Bambara groundnut	0.7	0.2	179	-24.3	–	86.5
	Maize-Dry bean	0.7	0.6	230	26.6	–	-0.6

4.0 Discussion

Canopy parameters (Leaf number, leaf area, plant height and tiller number) for maize landrace that contribute to LAI were not improved by supplementary irrigation or intercropping. This would suggest that these maize landrace parameters could be considered stable across different cropping systems and water regimes. The observed trend for LAI and TAW reflects advantages of intercropping under water limited conditions. The higher LAI observed under intercropped maize landraces was attributed to the added bambara groundnut and dry bean. Planting either bambara groundnut or dry bean in-between the maize rows reduced time to maximum canopy cover. As a result, the soil was only exposed to the drivers of evaporation (wind solar energy) for a shorter time during the growth period. It could be that, the intercropped plant species could have created a barrier from wind and solar radiation increasing relative humidity and decreasing canopy and soil surface temperature. This then resulted in a reduction in the evaporative demand for the immediate atmosphere around the canopy; thus reducing soil evaporation and increasing overall TAW. In this regard, the added crop species acted as a live-mulch and minimized soil water evaporation and changed the microclimate in the canopy. Improvements in LAI brought about by intercropping maize landraces with legumes can improve the availability of water for crop use under rainfed cropping systems.

The observed higher g_s, CCI and Fm/Fv for intercropped maize landrace grown under rainfed conditions suggest that intercropping can also increase photosynthetic efficiency of maize landrace. Improvements in leaf physiological response for intercropped maize landraces were attributed to improvements in TAW which were brought about by the observed increase in LAI. Growth and yielding potential of a plant are determined by how efficient it can capture and utilize resources. Under low TAW, similar to what was observed for sole cropped maize landrace, it could be that stomata aperture on the leaf surface of the maize landrace closed to reduce the loss of plant water through transpiration, this, in turn, reduced g_s and lowered the uptake of CO_2. Prolonged exposure to low TAW could have then resulted in the degradation of chlorophyll and a reduction in Fm/Fv. Under water stress, there is a reduction in the biosynthesis of chlorophyll and the functionality of the PSII system to accommodate for the downregulation of metabolic processes and photosynthetic reactions. In this

regard, the parameters CCI and Fm/Fv can be used to depict the state of photosynthetic apparatus within the leaf. Through the modification of eco-physiology, intercropping can improve photosynthetic efficiency of maize landrace in areas where is it grown under water-limited conditions.

The observed trend for biomass, yield and yield parameter for maize landrace are contrary to observed results of leaf physiology and TAW. It was expected that, the reduction in leaf photosynthetic efficiency as a result of low g_s, CCI and Fv/Fm would result in a reduction in CO_2 uptake and assimilation; concomitantly, biomass and yield. It could be that the magnitude of reduction for leaf physiology did not have a significant effect on leaf photosynthetic efficiency. It was observed that temperature for the 2015/16 summer growing season were higher than the long term average maximum and minimum temperatures. When plants are grown under well-watered conditions the rate of respiration has been observed to go up under warmer conditions resulting in a reduction in CO_2 assimilates and ultimately biomass. It could be that improving TAW for maize landraces also resulted in an increase in photo-respiration.

References

Aguiriano, E., Ruiz, M., Fité, R., Carrillo, J.M., 2008. Genetic variation for glutenin and gliadins associated with quality in durum wheat (Triticum turgidum L. ssp. turgidum) landraces from Spain. Spanish J. Agric. Res. 6, 599–609.

Akpalu, M., Sarkodie-Addo, J., Akpalu, S., 2012. Effect of Spacing on Growth and Yield of Five Bambara Groundnut (Vigna Subterranea (L) Verdc.) Landraces. J. Sci. Technol. 32, 9–19. doi:10.4314/just.v32i2.2

Allen, R., Pereira, L.S., Raes, D., Smith, M., 1998. Crop evapotranspiration: Guidelines for computing crop requirements, Irrigation and Drainage Paper No. 56, FAO. Irrigation and Drainage Paper No. 56, FAO.

Bazargani, M.M., Sarhadi, E., Bushehri, A.A.S., Matros, A., Mock, H.P., Naghavi, M.R., Hajihoseini, V., Mardi, M., Hajirezaei, M.R., Moradi, F., Ehdaie, B., Salekdeh, G.H., 2011. A proteomics view on the role of drought-induced senescence and oxidative stress defense in enhanced stem reserves remobilization in wheat. J. Proteomics 74, 1959–1973.

doi:10.1016/j.jprot.2011.05.015

Botha, J., Anderson, J., Staden, P. Van, 2015. Rainwater harvesting and conservation tillage increase maize yields in South Africa. Water Resour. Rural ….

Cairns, J.E., Hellin, J., Sonder, K., Araus, J.L., MacRobert, J.F., Thierfelder, C., Prasanna, B.M., 2013. Adapting maize production to climate change in sub-Saharan Africa. Food Secur. 5, 345–360. doi:10.1007/s12571-013-0256-x

Chikowo, R., Zingore, S., Snapp, S., Johnston, A., 2014. Farm typologies, soil fertility variability and nutrient management in smallholder farming in Sub-Saharan Africa. Nutr. Cycl. Agroecosystems 100, 1–18. doi:10.1007/s10705-014-9632-y

Chimonyo, V.G.P., 2016. Quantifying productivity and water use of sorghum intercrop systems. University of KwaZulu-Natal, Pietermaritzburg, South Africa.

Chimonyo, V.G.P.V.G.P.V.G.P., Modi, A.T.A.T., Mabhaudhi, T., 2015. Perspective on crop modelling in the management of intercropping systems. Arch. Agron. Soil Sci. 61, 1–19. doi:10.1080/03650340.2015.1017816

Driedonks, N., Rieu, I., Vriezen, W.H., 2016. Breeding for plant heat tolerance at vegetative and reproductive stages. Plant Reprod. 29, 67–79. doi:10.1007/s00497-016-0275-9

Gao, Y., Duan, A., Qiu, X., Li, X., Pauline, U., Sun, J., Wang, H., 2013. Modeling evapotranspiration in maize/soybean strip intercropping system with the evaporation and radiation interception by neighboring species model. Agric. Water Manag. 128, 110–119. doi:10.1016/j.agwat.2013.06.020

Hellin, J., Bellon, M.R., Hearne, S.J., 2014. Maize Landraces and Adaptation to Climate Change in Mexico. J. Crop Improv. 28, 484–501. doi:10.1080/15427528.2014.921800

Jensen, J.., Bernhard, R.., Hansen, S., McDonagh, J., Møberg, J.., Nielsen, N.., Nordbo, E., 2003. Productivity in maize based cropping systems under various soil–water–nutrient management strategies in a semi-arid, alfisol environment in East Africa. Agric. Water Manag. 59, 217–237. doi:10.1016/S0378-

3774(02)00151-8

Jun, F., Yu, G., Quanjiu, W., Malhi, S.S., Yangyang, L., 2014. Mulching effects on water storage in soil and its depletion by alfalfa in the Loess Plateau of northwestern China. Agric. Water Manag. 138, 10–16. doi:10.1016/j.agwat.2014.02.018

Kunz, R.P., Davis, N.S., Thornton-Dibb, S., Steyn, J.M., Jewitt, G., 2015. Assessment of biofuel feedstock production in South Africa: Atlas of water use and yield of biofuel crops in suitable growing areas (Volume 3) Report to the WATER RESEARCH COMMISSION.

Mabhaudhi, T., Chimonyo, V.G.P., Chibarabada, T.P., Modi, A.T., 2017. Developing a Roadmap for Improving Neglected and Underutilized Crops: A Case Study of South Africa. Front. Plant Sci. 8. doi:10.3389/fpls.2017.02143

Mabhaudhi, T., Modi, A.T., 2014. Intercropping Taro and Bambara Groundnut, in: Lichtfouse, E. (Ed.), Sustainable Agriculture Reviews, Sustainable Agriculture Reviews. Springer International Publishing, Cham, pp. 275–290. doi:10.1007/978-3-319-00915-5_9

Mabhaudhi, T., Modi, A.T., Beletse, Y.G., 2013. Growth, phenological and yield responses of a bambara groundnut (Vigna subterranea L. Verdc) landrace to imposed water stress: II. Rain shelter conditions. Water SA 39, 191–198. doi:10.4314/wsa.v39i2.2

Mazvimbakupa, F., 2014. Water use characteristics of selected South African maize (Zea mays L.) landraces compared with commercial hybrids. University of KwaZulu-Natal.

McMaster, G.S., Wilhelm, W.W., 1997. Growing degree-days: One equation, two interpretations. Agric. For. Meteorol. 87, 291–300. doi:10.1016/S0168-1923(97)00027-0

Meyer, F., Davids, T., Van der Westhuizen, D., Louw, M., Vermeulen, H., 2016. The impact of the drought on South African consumers and the agricultural sector. Oilseeds Focus 2, 26–28.

Modi, A.T., Mabhaudhi, T., 2013. Water use and drought tolerance of selected traditional and indigenous crops. Final Report of Water Research Commission Project K5/1771//4. ISBN 978-1-4312-0434-2. Water Research Commission. Pretoria, South Africa.

Naim, A.M. El, Kilali, B.A., Hassan, A.E., Ahmed, M.F., 2013. Agronomic Evaluation of Sorghum and Cowpea Intercropped at Different Spatial Arrangements. J. Renew. Agric. 1, 11–16. doi:10.12966/jra.05.01.2013

Ncube, B., Finnie, J.F., Van Staden, J., 2011. Seasonal variation in antimicrobial and phytochemical properties of frequently used medicinal bulbous plants from South Africa. South African J. Bot. 77, 387–396. doi:10.1016/j.sajb.2010.10.004

Nduku, H., 2014. Crop Performance and Soil Nutrient Dynamics under Different Organic Based Cropping Systems and Modeling The Effect of Climate Change On Maize (Zea May). University of Nairobi.

Oliveira, R.B.R. de, Moreira, R.M.P., Ferreira, J.M., 2013. Adaptability and stability of maize landrace varieties. Semin. Ciências Agrárias 34, 2555. doi:10.5433/1679-0359.2013v34n6p2555

Ortmann, G.F., King, R.P., 2010. Research on agri-food supply chains in Southern Africa involving small-scale farmers: Current status and future possibilities. Agrekon 49, 397–417. doi:10.1080/03031853.2010.526428

Sujatha, S., Bhat, R., 2010. Response of vanilla (Vanilla planifolia A.) intercropped in arecanut to irrigation and nutrition in humid tropics of India. Agric. Water Manag. 97, 988–994. doi:10.1016/j.agwat.2010.01.031

TerAvest, D., Carpenter-Boggs, L., Thierfelder, C., Reganold, J.P.J.P., 2015. Crop production and soil water management in conservation agriculture, no-till, and conventional tillage systems in Malawi 212, 285–296. doi:10.1016/j.agee.2015.07.011

Thrupp, L. a, 2000. Linking agricultural biodiversity and food security: the valuable role of agrobiodiversity for sustainable agriculture. Int. Aff. 76, 265–281. doi:10.1111/1468-2346.00133

Van Averbeke, W., Denison, J., Mnkeni, P., 2011. Smallholder irrigation schemes in South Africa: A review of knowledge generated by the Water Research Commission. Water SA 37, 797–808. doi:10.4314/wsa.v37i5.17

Van Averbeke, W., Khosa, T.B., 2007. The contribution of smallholder agriculture to the nutrition of rural households in a semi-arid environment in South Africa, in: Water SA. pp. 413–418. doi:10.4314/wsa.v33i3.49158

Wang, Z.-G., Jin, X., Bao, X.-G., Li, X.-F., Zhao, J.-H., Sun, J.-H., Christie, P., Li, L., 2014. Intercropping enhances productivity and maintains the most soil fertility properties relative to sole cropping. PLoS One 9, e113984. doi:10.1371/journal.pone.0113984

Wenhold, F.F., Faber, M., Averbeke, W. Van, Oelofse, A., Jaarsveld, P.. Van, Jansen van Rensburg, W., van Heerden, I., Slabbert, R., 2007. Linking smallholder agriculture and water to household food security and nutrition. WaterSA 33, 327–336.

Willey, R.., 1979. Intercropping-Its importance and research needs.1.Competition and yield advantages. F. Crop Abstr. 32, 1–10.

Wise, R., Cacho, O., Hean, R., 2007. Fertilizer effects on the sustainability and profitability of agroforestry in the presence of carbon payments. Environ. Model. Softw. 22, 1372–1381. doi:10.1016/j.envsoft.2006.10.002

Zeven, A., 1998. Landraces: a review of definitions and classifications. Euphytica.
